# Comparison between strip sampling and laser ablation methods to infer seasonal movements from intra-tooth strontium isotopes profiles in migratory caribou

**DOI:** 10.1038/s41598-023-30222-w

**Published:** 2023-03-03

**Authors:** Mael Le Corre, Vaughan Grimes, Rebecca Lam, Kate Britton

**Affiliations:** 1 Department of Archaeology, University of Aberdeen, Aberdeen, AB252SU UK; 2 Department of Archaeology, Memorial University of Newfoundland, St. John’s, NL A1C 5S7 Canada; 3 Department of Earth Sciences, Memorial University of Newfoundland, St. John’s, NL A1C 5S7 Canada; 4 CREAIT Network, Memorial University of Newfoundland, St. John’s, NL A1C 5S7 Canada

**Keywords:** Palaeoecology, Palaeoecology, Stable isotope analysis, Geochemistry

## Abstract

Strontium isotopes analysis is a powerful tool in the study of past animal movements, notably the sequential analysis of tooth enamel to reconstruct individual movements in a time-series. Compared to traditional solution analysis, high resolution sampling using laser-ablation multi-collector inductively coupled plasma mass spectrometry (LA-MC-ICP-MS) has the potential to reflect fine scale mobility. However, the averaging of the ^87^Sr/^86^Sr intake during the enamel mineralization process may limit fine scale inferences. We compared solution and LA-MC-ICP-MS ^87^Sr/^86^Sr intra-tooth profiles from the second and third molars of 5 caribou from the Western Arctic herd, Alaska. Profiles from both methods showed similar trends, reflecting the seasonal migratory movements, but LA-MC-ICP-MS profiles showed a less damped ^87^Sr/^86^Sr signal than solution profiles. Geographic assignments of the profile endmembers to the known summer and winter ranges were consistent between methods and with the expected timing of enamel formation but showed discrepancy at a finer scale. Variations on LA-MC-ICP-MS profiles, consistent with expected seasonal movements, suggested more than an admixture of the endmember values. However, more work in understanding enamel formation in *Rangifer*, and other ungulates, and how ^87^Sr/^86^Sr daily intake translates into enamel are needed to assess the real resolution that can be achieved with LA-MC-ICP-MS.

## Introduction

Strontium isotope analysis has proven to be a powerful tool for the reconstruction of past movements. Based upon the premise of a relationship between the strontium isotope chemistry in underlying local geology/local soils and skeletal tissues, these techniques are increasingly employed to investigate human and faunal movements in the past^1^. Studies have analysed faunal remains to infer trade and exchange networks^2,3^, and other aspects of past economies and societies, including transhumance, animal husbandry and herding practices^4,5^. Other archaeological and palaeontological studies have focused on wild taxa to explore seasonal animal palaeobiogeography, including the reconstruction of past migratory behaviours in extinct species^6,7^ and important prey-species, as a means to better understand the movement patterns, landscape use and subsistence decisions of human groups that depended on them^8–11^.

Strontium exists naturally as three stable isotopes ^84^Sr, ^86^Sr and ^88^Sr, and as a radiogenic isotope, ^87^Sr, which is the product of the radioactive decay of ^87^Rb (Rubidium). Because of the decay of ^87^Rb, ancient Rb-rich rocks such as potassium feldspar containing granite and rhyloite, are expected to have high ^87^Sr/^86^Sr while more recent lithologies and/or those poor in Rb like carbonates, have a lower ratio^12^. Via soil formation, strontium becomes available for plants, along with other inputs (e.g., sea-spray, rainfall, aeolian deposition) which can influence ‘bioavailable’ ^87^Sr/^86^Sr. Despite these other influences, the spatial distribution of the bioavailable ^87^Sr/^86^Sr across the landscape is mainly related to the nature and age of underlying lithological units^13,14^. Bioavailable ^87^Sr/^86^Sr is incorporated to mammalian tissues through food and water consumed. During the transfer process from bedrock to animal tissues, ^87^Sr/^86^Sr is subjected to negligible fractionation^15^ and thus, values observed in animal tissues should directly reflect the local bioavailable ^87^Sr/^86^Sr of the area where the animal fed during the tissue formation^13^, albeit with some potential influences of differential digestion or other aspects of food selection or preparation^16–18^.A recent effort has been made to produce local and global ^87^Sr/^86^Sr isoscapes based on intensive sampling of soil and biological material and/or modelling^14,19,20^. As ^87^Sr/^86^Sr varies in bedrock on a geological time scale, modern strontium isoscapes are relevant to explore past mobility, although anthropogenic influences that also affect environmental ^87^Sr/^86^Sr (e.g., fertilizers) should be taken into account^14,21^.

In mammals, after absorption in the gut, strontium is incorporated into bioapatite in bones and enamel through ionic substitution of calcium^12^. While bones are constantly renewed during the life of an individual, tooth enamel does not remodel once mineralized and, due to its structure, is particularly resistant to diagenesis^22^. Thus, ^87^Sr/^86^Sr values in tooth enamel can provide reliable geolocation information for individuals during their early stages of life. Studies have proven their usefulness to assess faunal mobility^7,10,23^, relying on the principle that mammalian tooth enamel forms incrementally from the cusp of the crown to the enamel-root junction, according to a two steps process^24^. During the secretion phase, a protein-rich matrix is produced following a secretion front toward the cervix of the tooth. Then, during the maturation phase, most of the mineralised material is incorporated to the enamel matrix^24^. This phase is complex and non-linear with a rapid initial mineralization rate that slows down progressively, spanning over weeks depending on the species^25^. However, mineralization broadly progresses along the growth axis following the secretion front^24–26^, allowing temporal inferences on the isotopic signal observed in the tooth. Comparison of bulk enamel ^87^Sr/^86^Sr with local ^87^Sr/^86^Sr allows to discriminate between local and non-local individuals^27^ but the analysis of intra-tooth ^87^Sr/^86^Sr values can highlight short-term mobility^7,22^.

The traditional approach to analyse strontium isotope ratios in tooth enamel is to cut a sample (e.g., hand drill, micro-mill) then to undertake anion exchange chemistry (solution method) to isolate and purify the strontium prior to analysis. During this process the sample is dissolved in high-purity acid, and Sr is isolated using a Sr Resin^28^. This is followed by analysis via thermal ionisation mass spectrometry (TIMS) or by multi-collector inductively coupled plasma mass spectrometry (MC-ICP-MS). Analyses can be done on bulk (whole tooth) enamel samples or on samples taken along the growth axis of an individual tooth^23,29^. Strontium isotope analysis can be combined with *ẟ*^18^O analysis to identify which sections of the tooth formed during cooler or warmer periods, placing the ^87^Sr/^86^Sr time-series data within a seasonal framework^9,29^. ^87^Sr/^86^Sr values generated can then be compared with strontium isoscapes, directly or using probabilistic approaches, in order to identify potential areas of origin or seasonal ranges^30,31^. A major drawback of solution analysis is the amount of material needed to conduct the analysis (∼ 1–5 mg) which averages ^87^Sr/^86^Sr intakes over weeks^26,32^. Moreover, this approach is destructive, limiting its application on rare archaeological and palaeontological specimens.

Alternatively, *in-situ* measurement using laser-ablation (LA)-MC-ICP-MS, allows high resolution sampling along the enamel surface with often less obvious alteration of the material^26,32^. While LA-MC-ICP-MS has been reported to produce less precise and less accurate ^87^Sr/^86^Sr data than solution analysis due to molecular/analytical ^87^Sr interferences stemming primarily from the formation of ^40^Ca^31^P^16^O^32,33^, the associated analytical errors can remain low enough to study geographic origin and mobility in many geological/environmental context^26,34^. Due to the high sampling resolution of LA-MC-ICP-MS, questions have arisen about its ability to reflect mobility habits on a more refined scale and the potential time resolution that can be achieved^26,35–37^. A main concern is to what extent the ^87^Sr/^86^Sr is averaged within the body before and during the mineralization process, regardless of how high-resolution sampling strategies might be. For example, in addition to the daily strontium intake (food, water), strontium is released during the continuous remodelling of bones contributing to blood strontium^38^, and inducing a ^87^Sr/^86^Sr baseline reflecting the average ^87^Sr/^86^Sr in bones. In ungulates most of the mineralization at any specific location occurs in only a few days before slowing down and then continuing over several weeks^25^. The real impact of these two averaging processes on the ability of intra-tooth ^87^Sr/^86^Sr profiles to reflect changes in location is unclear. Montgomery et al.^35^ suggested that intermediate values within any intra-tooth sequence are only the product of mixing the signal of the endmembers, the ^87^Sr/^86^Sr value of the new location overprinting gradually the original signal. However recent studies on human^26,37,39^ and sheep^36^ dental enamel have provided evidence of short-term variations in intra-tooth ^87^Sr/^86^Sr profiles that contradict an admixture of endmembers signal, and instead reflect more the movement of the individuals through the strontium landscape^36^. More studies are needed to fully assess the potential of LA-MC-ICP-MS in reconstructing faunal movements, notably in regard to the well-established solution analysis.

Using modern caribou (*Rangifer tarandus*) teeth from migratory animals, here we aim to compare solution and LA-MC-ICP-MS ^87^Sr/^86^Sr intra-tooth profiles from the same individuals. Caribou and reindeer are important taxa in northern environments, and both are central in the culture and subsistence of circumpolar indigenous communities^40–42^. There is a long history of *Rangifer* population exploitation by human societies, in North America, and throughout Eurasia. During the Late Pleistocene in north-west Europe, reindeer were at the centre of the economy and a herd’s seasonal movements likely shaped human subsistence practices and landscape use (i.e., lifeways), and even the cultures and worldviews of human groups^43^. Modern wild caribou demonstrate variability in their seasonal movements with resident and migratory ecotypes^40^ and within a given population, migration patterns can drastically change over a period of only a few years^44^. The extent to which there were diverse ecotypes within Late Pleistocene Europe, and/or the extent to which these behaviours were plastic or conservative through time remains poorly characterised. Reconstructing past mobility and seasonality of reindeer is therefore essential to understand landscape use and other cultural aspects of past human and hominin groups^8,10^, but also to understanding the palaeoecology of these species. The increased resolution offered by LA-MC-ICP-MS may help us better understand these. Furthermore, the usefulness of spatial assignment tools in empirically identifying summer and winter ranges from intra-tooth data (generated via solution or LA-MC-ICP-MS approaches) has yet to be tested on a modern, wild migratory *Rangifer*. The objectives of this study were therefore: (1) to generate novel intra-tooth ^87^Sr/^86^Sr data from five modern caribou from the Western Arctic herd, Alaska, a herd of well-documented seasonal movement pattern^45–47^, using LA-MC-ICP-MS, undertaken as a continuous raster down the full growth axis, (2) to compare the obtained LA-MC-ICP-MS intra-tooth ^87^Sr/^86^Sr profiles to already published intra-tooth profiles from the same individuals, generated by the ‘traditional’ strip sampling and solution method^23^, and (3) to compare the potential summer and winter range use through the spatial assignment of data from seasonal endmembers obtained from both the solution and LA-MC-ICP-MS sequences.

## Results

### ^87^Sr/^86^Sr intra-tooth profiles

**Figure 1:**
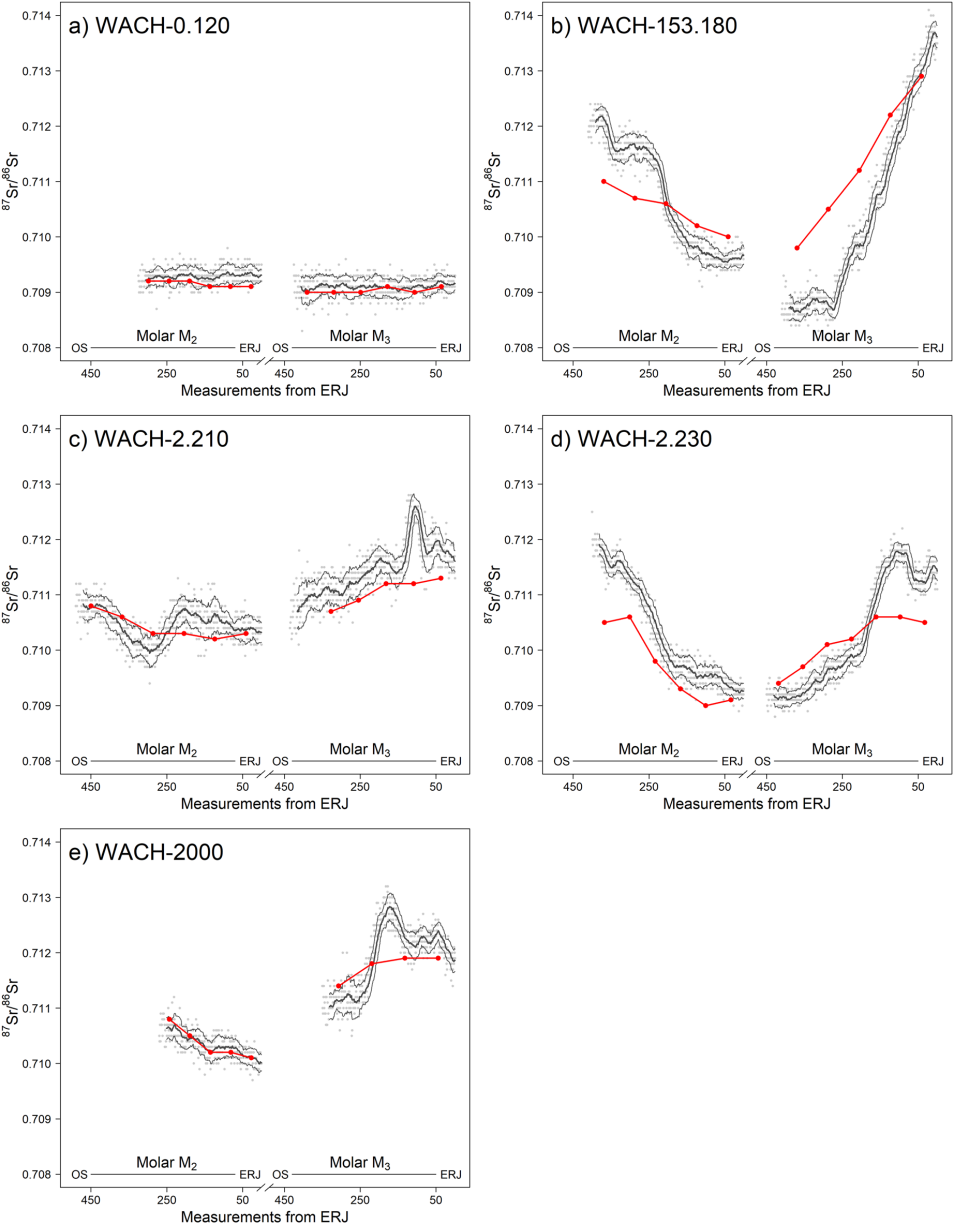
Solution (in red) and LA-MC-ICP-MS (in black) ^87^Sr/^86^Sr intra-tooth profiles of the M_2_ and M_3_ from 5 Western Arctic herd caribou. Solution profiles are reproduced from Britton et al.^23^. For the LA-MC-ICP-MS profiles the black line corresponds to the running average (± running standard deviation), based on a 20 samples window. The x-axis corresponds to the location of the LA-MC-ICP-MS samples from the enamel-root junction. Solution sample locations were adjusted to LA-MC-IPC-MS profiles only to facilitate visual comparison. Solution and LA-MC-ICP-MS values at a given location should not be directly compared as the two analyses were performed on opposite tooth surfaces or mirroring teeth. ERJ: Enamel-root junction, OS: Occlusal surface.

Using LA-MC-ICP-MS analysis, we generated ^87^Sr/^86^Sr intra-tooth profiles from the second (M_2_) and third molar (M_3_) of 5 caribou from the Western Arctic herd (Fig. 1). These profiles followed similar general trends to already published solution ^87^Sr/^86^Sr intra-tooth profiles from the same 5 individuals^23^, and taken from the same or mirroring M_2_ and M_3_ (Table S1). For 4 individuals we observed a decline in ^87^Sr/^86^Sr values from the oldest part of the crown (occlusal surface—OS) to the youngest part (enamel-root junction—ERJ) of the M_2_ and the opposite trend was observed on the M_3_ (Fig. 1). For one individual, WACH-0.120, ^87^Sr/^86^Sr values remained relatively stable along its M_2_ and M_3_ intra-tooth profiles (Fig. 1). Solution profiles presented a dampened signal compared to LA-MC-ICP-MS profiles. ^87^Sr/^86^Sr values ranged from 0.7090 to 0.7129 for solution profiles and from 0.7087 to 0.7137 for LA-MC-ICP-MS profiles (Table 1). Minimum ^87^Sr/^86^Sr values were almost significantly lower for LA-MC-ICP-MS (0.7096 ± 0.0008 SD) than for solution (0.7099 ± 0.0008 SD; Wilcoxon signed-rank test: *n* = 20, *Z* = 1.82, *P* = 0.08) and maximum ^87^Sr/^86^Sr values were significantly higher (respectively 0.7115 ± 0.0015 SD and 0.7108 ± 0.0011 SD; *n* = 20, *Z* = − 2.55, *P* < 0.05). However, median ^87^Sr/^86^Sr values in LA-MC-ICP-MS (0.7103 ± 0.0009 SD) and solution profiles (0.7103 ± 0.0009 SD) were similar (*n* = 20, *Z* = − 0.513, *P* = 0.64). LA-MC-ICP-MS profiles had a higher amplitude than solution profiles (respectively 0.0019 ± 0.0015 SD and 0.0010 ± 0.0009 SD; *n* = 20, *Z* = − 2.67, *P* < 0.01) and differences in amplitude between LA-MC-ICP-MS and solution profiles were positively correlated with LA-MC-ICP-MS amplitude (*R*^*2*^ = 0.81, *P* < 0.01).

**Table 1:** Descriptive statistics of the ^87^Sr/^86^Sr intra-tooth profiles of the M_2_ and M_3_ from 5 Western Arctic herd caribou assessed from solution analysis and LA-MC-ICP-MS analysis.

Individual	Molar	Solution				LA-MC-ICP-MS				Diff. amp
		Median	Min	Max	Amplitude	Median	Min	Max	Amplitude	
WACH-0.120	M_2_	0.7092	0.7091	0.7092	0.0001	0.7093	0.7092	0.7094	0.0002	0.0001
WACH-0.120	M_3_	0.7090	0.7090	0.7091	0.0001	0.7091	0.7090	0.7092	0.0002	0.0001
WACH-153.180	M_2_	0.7106	0.7100	0.7110	0.0010	0.7104	0.7095	0.7122	0.0026	0.0016
WACH-153.180	M_3_	0.7112	0.7098	0.7129	0.0031	0.7099	0.7087	0.7137	0.0050	0.0019
WACH-2.210	M_2_	0.7103	0.7102	0.7108	0.0006	0.7105	0.7099	0.7108	0.0009	0.0003
WACH-2.210	M_3_	0.7112	0.7107	0.7113	0.0006	0.7114	0.7107	0.7126	0.0019	0.0013
WACH-2.230	M_2_	0.7096	0.7090	0.7106	0.0016	0.7103	0.7092	0.7119	0.0027	0.0011
WACH-2.230	M_3_	0.7102	0.7094	0.7106	0.0012	0.7099	0.7091	0.7118	0.0027	0.0015
WACH-2000	M_2_	0.7102	0.7101	0.7108	0.0007	0.7103	0.7100	0.7107	0.0007	0.0000
WACH-2000	M_3_	0.7119	0.7114	0.7119	0.0005	0.7122	0.7110	0.7128	0.0018	0.0013

diff. amp: difference between the amplitude of the solution and the LA-MC-ICP-MS profiles.

To set seasonality in ^87^Sr/^86^Sr intra-tooth profiles, we relied and on the *ẟ*^13^C and *ẟ*^18^O intra-tooth profiles from the same individuals (Figs. S1–S2), assessed in former studies^23,48^ and on the amelogenesis in fallow deer *Dama dama*^10,23^ occurring between 3.5 and 9 months for the M_2_ and between 9 and < 18 months for the M_3_^49^. For the 5 individuals, on the endmember of the M_2_ close to the OS, we considered the relatively low *ẟ*^13^C values (Fig. S1) and high *ẟ*^18^O values (Fig. S2), respectively synonymous with grass-rich diet^50,51^ and warm season^9,52^, indicative of the late summer/autumn period. Conversely, on the endmember close to the ERJ, we considered the high *ẟ*^13^C values and the low *ẟ*^18^O values, respectively synonymous with lichen-rich diet^50,51^ and cold season^9,52^, indicative of winter. The opposite was observed on the M_3_ (Figs. S1–S2), with the OS endmember indicative of the winter period and the ERJ endmember indicative of the late summer/autumn period. ^87^Sr/^86^Sr values of winter endmembers (M_2_-ERJ, M_3_-OS) were similar between LA-MC-ICP-MS and solution analyses (Table 2; respectively 0.7097 ± 0.0008 SD and 0.7099 ± 0.0008 SD; *n* = 20, *Z* = 1.05, *P* = 0.33) but summer endmembers (M_2_-OS, M_3_-ERJ) ^87^Sr/^86^Sr values from LA-MC-ICP-MS analysis were significantly higher (respectively 0.7112 ± 0.0013 SD and 0.7108 ± 0.0011 SD; *n* = 20, *Z* = − 2.03, *P* = 0.05). Such as for the total amplitude of the profiles, amplitude between endmembers from LA-MC-ICP-MS profiles were higher than in solution profiles (respectively 0.0015 ± 0.0015 SD and 0.0009 ± 0.0009 SD; *n* = 20, *Z* = − 2.2, *P* < 0.05).

**Table 2:** ^87^Sr/^86^Sr values of the endmembers from the solution and LA-MC-ICP-MS intra-tooth profiles of the M_2_ and M_3_ from 5 Western Arctic herd caribou.

Individual	Molar	Solution			LA-MC-ICP-MS			Diff. amp
		Occlusal surface	Enamel-root junction	Amplitude	Occlusal surface	Enamel-root junction	Amplitude	
WACH-0.120	M_2_	0.7092	0.7091	0.0001	0.7092	0.7093	0.0001	0.0000
WACH-0.120	M_3_	0.7090	0.7091	0.0001	0.7091	0.7092	0.0001	0.0000
WACH-153.180	M_2_	0.7110	0.7100	0.0010	0.7121	0.7097	0.0024	0.0014
WACH-153.180	M_3_	0.7098	0.7129	0.0031	0.7087	0.7136	0.0049	0.0018
WACH-2.210	M_2_	0.7108	0.7103	0.0005	0.7108	0.7103	0.0005	0.0000
WACH-2.210	M_3_	0.7107	0.7113	0.0006	0.7107	0.7116	0.0009	0.0003
WACH-2.230	M_2_	0.7105	0.7091	0.0014	0.7119	0.7093	0.0026	0.0012
WACH-2.230	M_3_	0.7094	0.7105	0.0011	0.7092	0.7114	0.0022	0.0011
WACH-2000	M_2_	0.7108	0.7101	0.0007	0.7106	0.7100	0.0006	– 0.0001
WACH-2000	M_3_	0.7114	0.7119	0.0005	0.7110	0.7119	0.0008	0.0003

Endmembers correspond to the closest samples from the occlusal surface and from the enamel-root junction. diff. amp: difference between the endmember amplitude of the solution and the LA-MC-ICP-MS profiles.

### Spatial assignment

**Figure 2:**
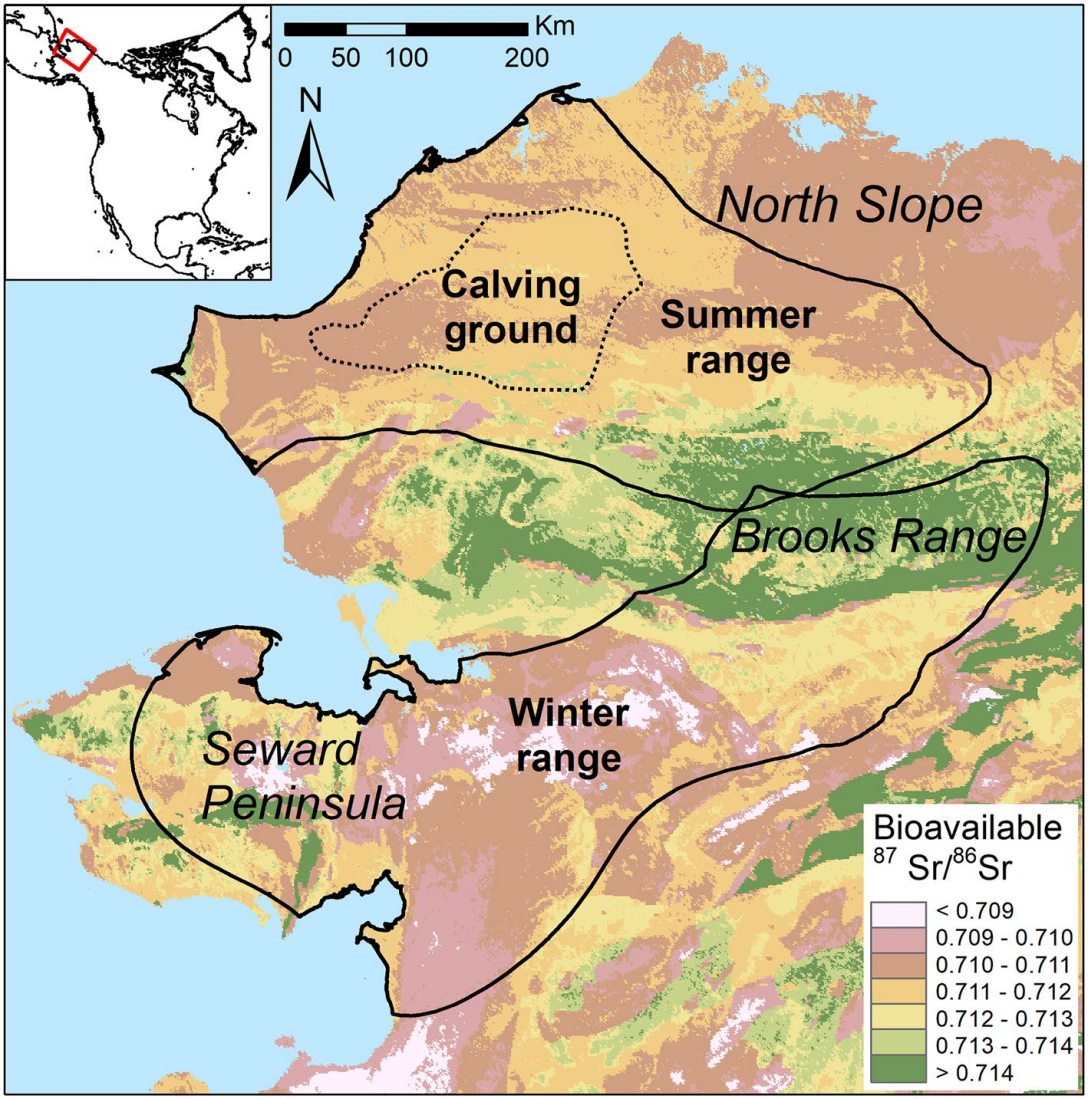
Bioavailable ^87^Sr/^86^Sr isoscape of Alaska. The map was created in ArcGIS 10.5 using data from the global bioavailable ^87^Sr/^86^Sr isoscape provided in Bataille et al.^14^. Summer range, winter range and calving ground of the Western Arctic migratory caribou herd are based on the telemetric survey of the herd by the Alaska Department of Fish and Game from 2004 to 2014^55^.

**Figure 3:**
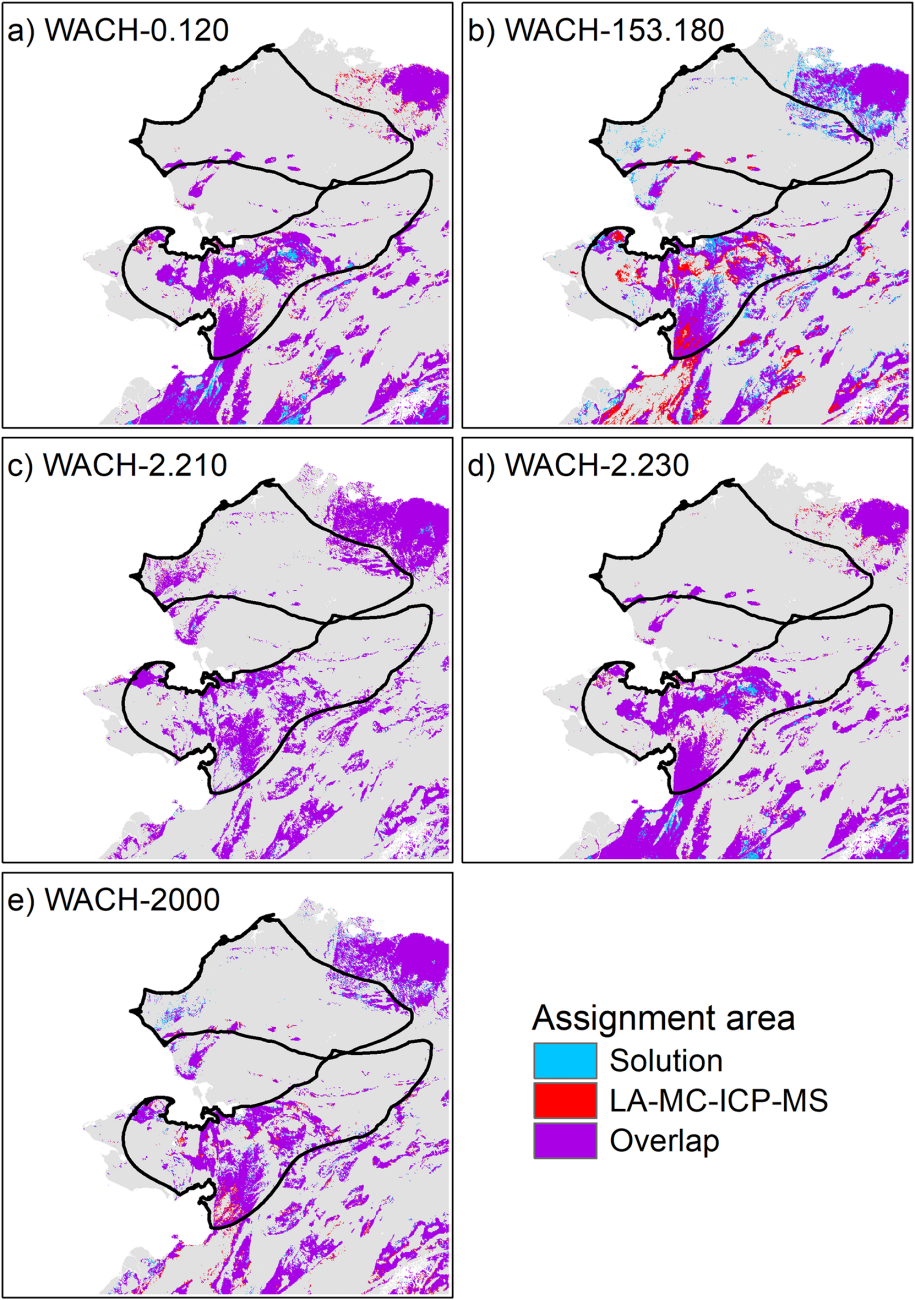
Overlap between the winter ^87^Sr/^86^Sr spatial assignment assessed from solution and LA-MC-ICP-MS ERJ endmember values of the M_2_ (enamel-root junction sample) for 5 caribou from the Western Arctic herd. Based on estimated timing of tooth enamel formation, the M_2_ tooth section close to the enamel-root junction is expected to mineralize in winter. Assignment areas correspond to the 20% of the map with the highest posterior probability. Summer (north) and winter (south) ranges are delineated in black. The maps were generated in R (v4.2.1) and formatted in ArcGIS 10.5.

**Figure 4:**
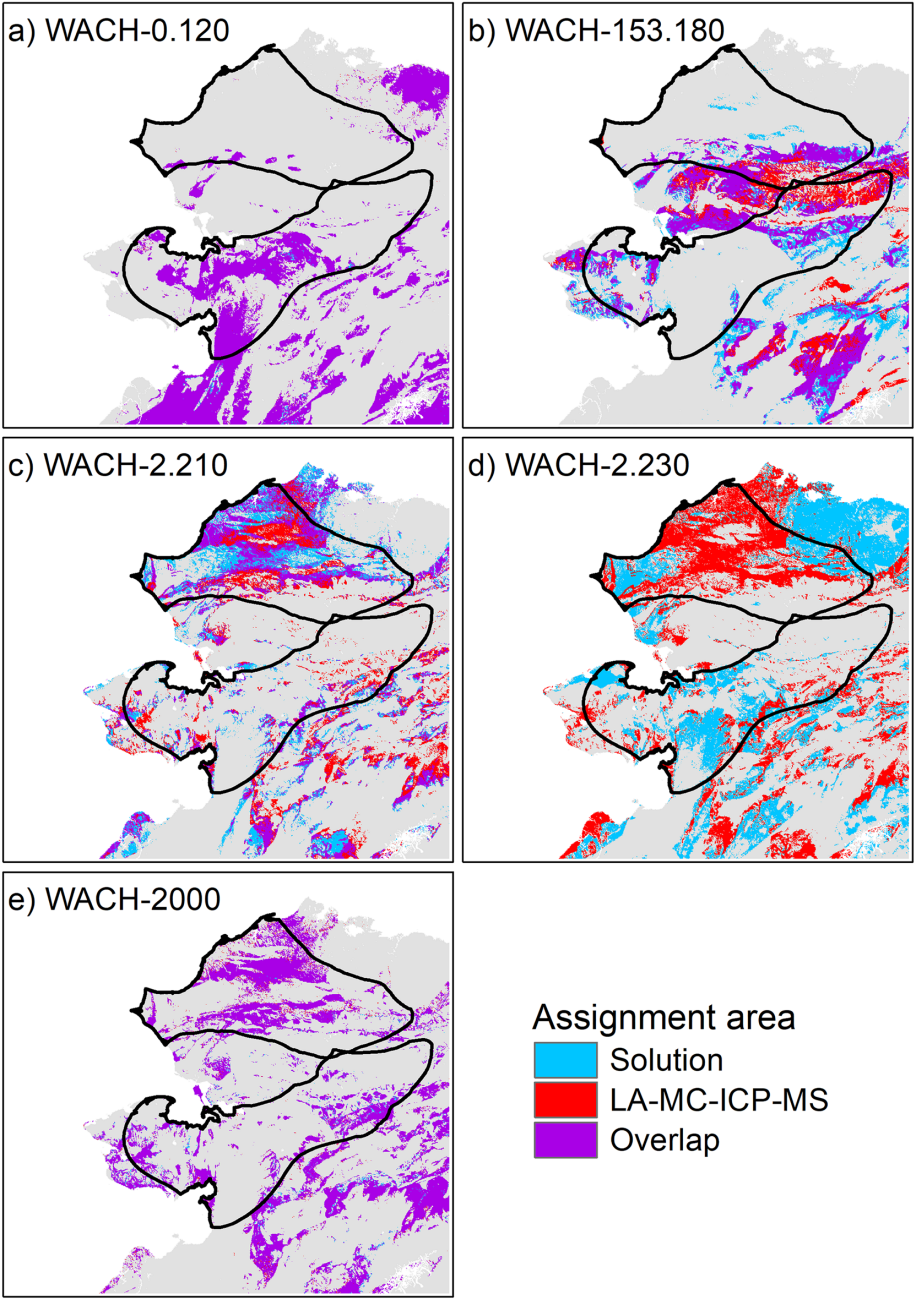
Overlap between the summer ^87^Sr/^86^Sr spatial assignment assessed from solution and LA-MC-ICP-MS ERJ endmember values of the M_3_ (enamel-root junction sample) for 5 caribou from the Western Arctic herd. Based on estimated timing of tooth enamel formation, the M_3_ tooth section close to the enamel-root junction is expected to mineralize in late summer/autumn. Assignment areas correspond to the 20% of the map with the highest posterior probability. Summer (north) and winter (south) ranges are delineated in black. The maps were generated in R (v4.2.1) and formatted in ArcGIS 10.5.

We performed Bayesian spatial assignment of the M_2_ and M_3_ endmembers to determine potential summer and winter ranges, using the assignR package^53^ in R^54^ with the global bioavailable ^87^Sr/^86^Sr isoscape from Bataille et al.^14^ cropped to north-west Alaska (Fig. 2). Overall bioavailable ^87^Sr/^86^Sr on the study area varied from 0.7075 to 0.7187 (95% reference interval) with extreme values of 0.7062 and 0.7271 (Fig. 2). We estimated the posterior probability of assignment of each endmember from ^87^Sr/^86^Sr solution and LA-MC-ICP-MS profiles, and extracted the corresponding assignment area, i.e., the 20% surface of the study area with the highest posterior probability of assignment. Posterior probability maps and assignment areas are provided in supplementary material for each individual (Figs. S3–S12). When comparing the posterior probabilities for known winter and summer ranges^55^ with odd ratios, most of the endmembers were assigned to the expected seasonal range (Table 3). All winter endmembers (M_2_-ERJ, M_3_-OS) were assigned to the southern range except for the M_3_-OS of two individuals, both for solution and LA-MC-ICP-MS profiles (Table 3; Fig. 3). Aside from the non-migratory caribou, WACH-0.120, that remained on the southern range (Table 3; Fig. 4), summer endmembers (M_2_-OS, M_3_-ERJ) were mostly assigned to the known summer range in the north (Table 3). The exception was the M_3_-ERJ endmember of WACH-153.180 assigned to the southern range. However, the corresponding assignment area, both for solution and LA-MC-ICP-MS analyses, fell mostly in the Brooks Range, in between the traditional summer and winter ranges (Fig. 4). Winter endmember assignment areas showed a strong agreement between solution and LA-MC-ICP-MS profiles (Fig. 3) with an overlap ranging from 30 to 99% (Table 3). Overlap was weaker for the summer assignment areas with no overlap observed in three occasions (Table 3), notably for WACH-2.230 (Fig. 4), despite all the corresponding endmembers being assigned, at the broad scale, to the traditional summer range.

**Table 3:** Comparison between the spatial assignment of the endmembers from the ^87^Sr/^86^Sr intra-tooth profiles of the M_2_ and M_3_ from 5 Western Arctic herd caribou assessed from solution analysis and LA-MC-ICP-MS analysis.

Individual	Molar	Sample	Season	Solution			LA-MC-ICP-MS			Overlap (%)
				Odd ratio	95% CI	Assigned range	Odd ratio	95% CI	Assigned range	
WACH.0.120	M_2_	OS	Summer	0.23	0.225–0.235	Winter	0.24	0.232–0.242	Winter	0.99
		ERJ	Winter	0.20	0.195–0.204	Winter	0.27	0.265–0.276	Winter	0.91
	M_3_	OS	Winter	0.17	0.17–0.178	Winter	0.20	0.195–0.204	Winter	0.98
		ERJ	Summer	0.20	0.195–0.204	Winter	0.22	0.211–0.221	Winter	0.98
WACH.153.180	M_2_	OS	Summer	1.63	1.599–1.658	Summer	1.58	1.553–1.611	Summer	0.00
		ERJ	Winter	0.70	0.685–0.711	Winter	0.46	0.45–0.468	Winter	0.73
	M_3_	OS	Winter	0.54	0.533–0.554	Winter	0.12	0.121–0.128	Winter	0.55
		ERJ	Summer	0.98	0.959–0.995	Winter	0.70	0.685–0.711	Winter	0.53
WACH.2.210	M_2_	OS	Summer	1.46	1.435–1.488	Summer	1.49	1.466–1.521	Summer	0.90
		ERJ	Winter	0.97	0.953–0.989	Winter	0.99	0.972–1.009	NS	0.97
	M_3_	OS	Winter	1.37	1.343–1.393	Summer	1.36	1.338–1.388	Summer	0.99
		ERJ	Summer	1.79	1.758–1.824	Summer	1.81	1.779–1.844	Summer	0.42
WACH.2.230	M_2_	OS	Summer	1.17	1.148–1.191	Summer	1.69	1.657–1.719	summer	0.00
		ERJ	Winter	0.20	0.195–0.204	Winter	0.25	0.247–0.259	Winter	0.94
	M_3_	OS	Winter	0.31	0.303–0.315	Winter	0.22	0.218–0.229	Winter	0.87
		ERJ	Summer	1.17	1.148–1.191	Summer	1.82	1.786–1.852	Summer	0.00
WACH.2000	M_2_	OS	Summer	1.46	1.435–1.488	Summer	1.30	1.28–1.328	Summer	0.66
		ERJ	Winter	0.78	0.77–0.799	Winter	0.71	0.697–0.724	Winter	0.91
	M_3_	OS	Winter	1.81	1.782–1.848	Summer	1.65	1.62–1.68	Summer	0.30
		ERJ	Summer	1.69	1.663–1.725	Summer	1.72	1.685–1.748	Summer	0.93

The likelihood of each sample to originate from either the winter or summer range was estimated from odd ratio (probability of assignment summer range/probability of assignment winter range), samples with an odd ratio > 1 being more likely to come from the summer range. Overlap (%) between the areas corresponding to 20% of the study area with the highest posterior probability assessed from the two sampling methods are provided. OS: occlusal surface, ERJ: enamel-root junction.

## Discussion

We compared the solution and LA-MC-ICP-MS ^87^Sr/^86^Sr intra-tooth profiles of 5 caribou from the Western Arctic herd (WAH) and assessed how differences in endmembers ^87^Sr/^86^Sr values of the M_2_ and M_3_ affected the summer and winter geographic assignment of these individuals. Among the 5 individuals, 4 displayed variability in their ^87^Sr/^86^Sr intra-tooth profiles, reflecting a migratory behaviour. Direct comparison of the output of the solution and LA-MC-ICP-MS methods requires samples taken at the same location on the tooth, accounting for the larger sampling site for solution analysis^26^. Here, the initial solution samples were destroyed during the analytical process^23^. When possible, we used the same tooth, on the lingual surface but for several teeth we had to rely on the molar from the other mandible. Crown height may differ between the inner and outer surface of a tooth, and between teeth of the left and right mandible. Left and right teeth can also present different levels of attrition^56^. Consequently, samples taken in different teeth or tooth surface should reflect an equivalent but not identical period of time. Thus, we limited our comparison between solution and LA-MC-ICP-MS profiles to global trends and to endmembers values.

The first difference observed between the solution and the LA-MC-ICP-MS ^87^Sr/^86^Sr intra-tooth profiles was on the amplitude between endmember ^87^Sr/^86^Sr values, solution profiles showing a dampening of the signal compared to LA-MC-ICP-MS. Due to the long mineralisation phase, the isotope input signal is time-averaged during amelogenesis leading to a natural attenuation of the signal within the tooth^24,57^. The strength of the attenuation is proportional to the maturation length, with greater attenuation for a longer time of maturation^24^. In the case of strontium, baseline blood values influenced by the long-term remodelling of bone also have the potential to mitigate the input signal^38^. Difference in attenuation between solution and LA-MC-ICP-MS analyses is likely due to sampling strategies^58^. Solution analysis was done on 1.5 mm wide strip samples taken on the entire thickness of the enamel. Enamel matures at different rates in different parts of the crown, with a slower rate of maturation close to the enamel-dentine junction^25,57^. Solution samples are likely to have a more extensively ‘averaged’ ^87^Sr/^86^Sr signal over a greater section of enamel than LA-MC-ICP-MS samples, spanning a longer maturation period, leading to a stronger dampening^58^. Moreover, the amplitude of LA-MC-ICP-MS ^87^Sr/^86^Sr variations were more consistent with the variations observed on the bioavailable ^87^Sr/^86^Sr isoscapes within the WAH range. Only for WACH-0.120, an individual that did not display migratory behaviour in either intra-tooth, do the solution and LA-MC-ICP-MS profiles demonstrate no discrepancy from one another. This suggests that when individuals are exposed to a constant, stable, bioavailable ^87^Sr/^86^Sr during the whole period of tooth growth both approaches should lead to similar output with no evidence of attenuation. Methods have been developed to retrieve the input signal from the observed intra-tooth profile for *ẟ*^13^C and *ẟ*^18^O accounting for attenuation^57^, and such approaches could be used for ^87^Sr/^86^Sr. However, it requires a good knowledge of the mineralisation process, notably of the apposition and the maturation length. Such parameters are known for several hypsodont ungulates (e.g., horse^59^, sheep^60^) but far less studied in brachydont ungulates^61^ and remain poorly characterised for *Rangifer.*

Despite differences in endmember values, LA-MC-ICP-MS ^87^Sr/^86^Sr intra-tooth profiles followed similar trends to solution profiles with a decline in ^87^Sr/^86^Sr signal while progressing toward the ERJ for the M_2_ and an increase for the M_3_, in the four migratory individuals, and a stable, low signal in the likely sedentary individual. Geographic assignments from LA-MC-ICP-MS and solution analyses appear to confirm the interpretation made on the solution ^87^Sr/^86^Sr intra-tooth profiles in the original study^23^. Relying on geological maps to interpret the variation in solution ^87^Sr/^86^Sr intra-tooth profiles, the decline in ^87^Sr/^86^Sr values observed in the M_2_ in this first study was attributed to an autumn seasonal movement from the summer range in the north within a high ^87^Sr/^86^Sr geological context, relative to this part of Alaska, toward the winter range in the south, within a lower ^87^Sr/^86^Sr geological context^23^. The opposite was observed on the M_3_, echoing the spring migration. In the current study, broad scale assignment to the known seasonal ranges using solution and LA-MC-IC-MS endmembers led to the same conclusions. However, on a finer scale, through the use of the assignment maps, differences on the potential location within a given seasonal range became evident between the two approaches. For the M_2_, the OS endmembers of the ^87^Sr/^86^Sr profiles were assigned to the summer range for all migratory caribou, while the ERJ endmembers were assigned to the winter range for all but one individual. For their M_3_, the OS endmembers were assigned to the winter range for half of the caribou and the ERJ endmembers of 3 individuals were assigned to the summer range and one belonged to the winter range. The sedentary individual, WACH-0.120, was always assigned to the southern range. Assignments were more nuanced when looking at the assignment maps. While low ^87^Sr/^86^Sr values (< 0.710) clearly designate the use of the southern range in winter, values between 0.710 and 0.711 could either indicate the southern and the northern areas. Higher ^87^Sr/^86^Sr values (> 0.7125) were mostly assigned to the Brooks Range in between the summer and the winter ranges. Migratory caribou are highly philopatric to their calving ground^46,62^ but show less fidelity to their winter range^44,63^. Caribou from WAH mainly winter south of the Brooks Range but can use areas further north with a higher ^87^Sr/^86^Sr signature^63^. Late summer movements are also highly variable in WAH with caribou using the northern range but also the Brooks Range^63^ within areas of medium to high bioavailable ^87^Sr/^86^Sr. OS endmembers should be taken with caution as they may have been exposed to wear as suggested notably by caribou WACH-2000, which had the lowest crown height. In this case the high ^87^Sr/^86^Sr value at the beginning of the M_3_ might not reflect the winter range ^87^Sr/^86^Sr signature, but rather a slightly later period and thus the transition toward the summer range. Despite a different degree of attenuation, solution and LA-MC-ICP-MS approaches appeared to reliably discriminate between potential seasonal ranges. However, to go beyond the assessment of broad scale origin a more refined sampling strategy, not normally possible for solution analysis due to sampling size requirements, is needed.

LA-MC-ICP-MS ^87^Sr/^86^Sr intra-tooth profiles showed peaks and troughs not visible on solution profiles. While transition from one endmember to the other occurred gradually on solution profiles, LA-MC-ICP-MS analysis highlighted clear sigmoid trends with steeps changes in ^87^Sr/^86^Sr values. Several teeth recorded small-scale increasing and declining phases within the global trend of the profile. Comparing ^87^Sr/^86^Sr cow’s molar profiles from solution-TIMS and LA-MC-ICP-MS analyses, Montgomery et al.^35^ concluded that intermediate values were the result of endmembers admixture and as such the higher resolution of LA-MC-ICP-MS analyses cannot be used to infer fine-scale movements. This was attributed to the long-term time-averaging mineralization process, meaning that a sampled area of tooth will incorporate strontium from multiple and not necessarily entirely synchronous events, and that during maturation strontium may even be incorporated more randomly in the crown, mitigating the signal^35^. However, measurements on human teeth have shown that minimum and maximum ^87^Sr/^86^Sr values from several LA-MC-ICP-MS profiles were outside of the range of the values observed at the beginning and at the end of these profiles, which does not support a simple admixture model of those endmembers^26^. This has also been observed in the current study. These differences may be attributed to the different tooth types of the species involved, and the different rates of enamel formation and mineralisation. While maturation of the enamel at a given location of a tooth can span a number of months, much of the process occurs in a short period of time before slowing down^25^. Thus, the resolution that can be achieved with LA-MC-ICP-MS analysis should likely depend on the duration of this fast mineralisation phase. In Mongolian caprines, relative changes in LA-MC-ICP-MS ^87^Sr/^86^Sr intra-tooth values reflected the use of pastures for relatively short periods of 3–4 weeks, indicating that for that species it may be possible to capture monthly movements^36^. Migratory caribou are a highly mobile species that can migrate over hundreds of kilometres, and are continuously moving, except for the wintering period and, for females and neonates, during calving^63,64^. High movement rates, particularly during migration, that could cover isotopically diverse areas over a short time frame may greatly limit the ability of the amelogenesis to capture that environmental variation in bioavailable ^87^Sr/^86^Sr compared to a singular ‘on–off’ movement between two isotopically distinct ranges. However, in the case of the WAH, caribou take several weeks to migrate across the Brooks Range^63^, an area separating the winter and the summer ranges and which presents the highest ^87^Sr/^86^Sr values in the WAH annual range (Fig. 2). Caribou may not spend enough time in the Brooks Range for their co-forming tissues to reach ^87^Sr/^86^Sr equilibrium, but this crossing is visible on the third molar with the increase in the ^87^Sr/^86^Sr followed by a decrease once they arrived on the calving ground. On the second molars we observed two patterns: a sigmoid from high to low ^87^Sr/^86^Sr and a more linear decrease from medium to low ^87^Sr/^86^Sr. Timing of autumn migration is highly variable with individuals already south of Brooks Ranges in late September while others are still crossing it in October^45,63^. The two different profiles could reflect this difference in the timing of Brooks Range crossing. While geographic assertion from intra-tooth profiles requires individuals to spend enough time in a homogeneous ^87^Sr/^86^Sr context^35^, small variations induced by short term residency within areas of specific ^87^Sr/^86^Sr signature, can be informative of landscape use. However, we have to remain very cautious with our inferences from the WAH caribou ^87^Sr/^86^Sr profiles as the main limit of our study remains the lack of knowledge about the precise timing of enamel mineralisation in *Rangifer*.

Estimations of the timing of dental formation in *Rangifer* is mostly based on data from fallow deer with the M_2_ forming between 3.5 and 9 months, and the M_3_ between 9 and 18 months^49^. According to modern *Rangifer* calving season, May/early June^46,65^, the M_2_ should mineralise from late summer to the first winter of life, encompassing the autumn migration. Intra-tooth profiles of *ẟ*^13^C and *ẟ*^18^O (Figs. S1–S2) are consistent with this expected timing of enamel formation^9,23,50,51^. The M_3_ should mineralised between the first and second winter of life, encompassing spring and autumn seasonal movements. However, ^87^Sr/^86^Sr (Fig. 2) and *ẟ*^13^C and *ẟ*^18^O intra-tooth profiles (Figs. S1–S2) suggest a shorter period of mineralisation with only one clear seasonal variation and eventually the beginning of the autumn. Alternatively, the end of the signal could be contracted, due to a slowing rate of mineralisation as observed in other ungulates^25,66^, averaging the isotopic intake of the last months into a smaller enamel section. Without a clear species-specific understanding of the timing of enamel formation, the seasonality in ^87^Sr/^86^Sr intra-tooth profiles should be inferred with caution and ideally when a seasonal context can be set, notably through *ẟ*^18^O isotope analysis.

Consistent with recent studies on sheep^36^ and humans^26^, the LA-MC-ICP-MS ^87^Sr/^86^Sr intra-tooth profiles of caribou we obtained here suggest that intra-tooth strontium isotope profiles generated in this way reveal more information than a simple admixture of the endmember signatures. However, further work linking bioavailable ^87^Sr/^86^Sr intake with the ^87^Sr/^86^Sr of intra-tooth profiles is needed to determine the real temporal resolution that can be achieved with LA-MC-ICP-MS analysis, and to assess how the magnitude of the variations in bioavailable ^87^Sr/^86^Sr reflects in the teeth. Controlled feeding studies are particularly useful to determine the timing of incorporation^24,67^ but may fail to translate the average intake of the different sources of strontium in natural conditions. Animal movement studies, such as Lazzerini et al.^36^, offer such opportunities but require a control of the date of birth of the sampled individuals, a continuous monitoring of their movements, and an accurate bioavailable ^87^Sr/^86^Sr isoscape to rely on. Studies on domestic or semi-domestic animals are likely more feasible than on wild population, due to the need to harvest the animals, and would allow, for example, to manipulate the time spent on isotopically distinct pastures at different locations^37^. Anchoring winter and summer periods with *ẟ*^18^O^9,52^ also provides valuable information, allowing the identification of potential seasonal ranges, and intermediate plateaus, peaks or troughs could be used to enlighten peculiar geological formations crossed during migration with a ^87^Sr/^86^Sr signature strong enough to influence intra-tooth profiles. Higher-resolution sampling and analysis for oxygen isotope analysis (e.g., using SHRIMP^68^), would be advantageous in more effectively linking intra-tooth oxygen and strontium profiles. Other approaches, such movement and habitat suitability models used in modern animal ecology to predict seasonal landscape use and migration routes from environmental features^45,69^ could also have potential to improve inferences to be made from intra-tooth isotopic datasets from ungulates. A recent study combined high resolution sampling of ^87^Sr/^86^Sr with movement modelling to reconstruct the lifetime movement of a mammoth from its tusk^7^. Applications could be extended through the determining of potential migration routes between seasonal ranges identified from intra-tooth profiles through the incorporation of landscape features that constrain or ease movements. Through accounting for bioavailable ^87^Sr/^86^Sr variations in the landscape during migration, the integration of such approaches with intra-tooth LA-MC-ICP-MS data from archaeological and palaeontological specimens would greatly improve our ability to reconstruct the mobility of past animals and—through this—better understand past human societies.

## Methods

### Western Arctic Herd (WAH) and samples

Caribou from the WAH inhabit a range of over 360,000 km^2^ in north-west Alaska. Main wintering areas are located south of the Brooks Range in the Nutalo Hills and Seward Peninsula (Fig. 2), while caribou spend the summer within the North Slope and the northern foothill of the Brooks Range^63^. The calving ground is located in Utukok uplands, in the western part of the North Slope. Traditional seasonal movements consist of the spring migration in April–May across the Brooks Range, arriving at the calving ground by early June. In early summer, during the insect relief season, caribou move to habitats that limit insect harassment, then they spread out to the wider summer range during the late summer period^63^. In autumn, from September to November, caribou migrate south to return to their winter range^63^. The summer range is predominantly dominated by lower and upper Cretaceous sedimentary continental rocks and conglomerates, the Brooks Range by Paleozoic sedimentary rocks and the winter range consists of Quaternary and Tertiary mafic volcanics rocks with some areas of Precambrian and Palaeozoic marine sedimentary rocks^70^.

We analysed teeth (M_2_ and M_3_) from 5 individuals (3 females, 2 males) provided by the Alaska Department of Fish and Game. Animals were part of the telemetric survey of the WAH^23^ and died from natural causes or harvested for purposes unrelated to this study. Each individual was estimated to be 5–9 years old and died within 10 years of each other but were unlikely to come from the same cohort. Following Animal Care policies, the sampled caribou were not equipped with radio-collars before they were at least 2 years old and after teeth formation. Thus, individual telemetric data cannot be used to infer ^87^Sr/^86^Sr intake and to correlate it with ^87^Sr/^86^Sr intra-tooth profiles, instead movements are inferred from the wider movements of the herd as a whole. Solution analysis of the M_2_ and M_3_ was done in a previous study^23^ and the LA-MC-ICP-MS analysis was conducted on the same teeth where possible, or from the opposing tooth where this was not possible (i.e., where teeth had been destroyed during the solution analysis process). LA-MC-ICP-MS analysis was conducted on the lingual surface, as opposed to the buccal surface for solution analysis, for the same reasons. Teeth presented different degrees of wear with crown height varying between 7.1 and 10.9 mm for the M_2_, and between 8.1 and 10.9 mm for the M_3_.

### Seasonality in teeth

In order to infer the seasonal variations in ^87^Sr/^86^Sr intra-tooth profiles we needed to define the seasonal context of the enamel formation. The timing of mineralisation in *Rangifer* is poorly characterised but using fallow deer teeth formation as a proxy^10,23^, M_2_ and M_3_ enamel formation are expected to occur between 3.5 and 9 months, and between 9 and < 18 months, respectively^49^. We also used *ẟ*^13^C and *ẟ*^18^O isotopes to determine seasonality^9,51^. Fluctuations in *ẟ*^18^O intra-tooth values reflect seasonal variations in temperatures with high *ẟ*^18^O values in summer and low *ẟ*^18^O values in winter^9,52^. On the other hand, variations in *ẟ*^13^C reflect changes in diet^51,67^. Caribou feed mainly on grass and vascular plants in summer then switch to a lichen-rich diet in winter^71^, which should translate into an enrichment in ^13^C^50^^,^^51^. Along with the initial ^87^Sr/^86^Sr analysis, sequential *ẟ*^13^C enamel data^48^ and sequential *ẟ*^18^O enamel data^23^ were produced for the 5 individuals through analysis of the carbonate moiety. We used these *ẟ*^13^C and *ẟ*^18^O profiles to anchor seasonality within the M_2_ and the M_3_, assuming high *ẟ*^13^C and *ẟ*^18^O low for winter and the opposite for summer. From these profiles and according to other *ẟ*^13^C and *ẟ*^18^O studies on modern and archaeological *Rangifer*^9,10^, we assumed, for the M_2_, the endmember close to the occlusal surface (OS) to correspond to the late summer/autumn period, and the endmember close to the enamel-root junction (ERJ) to correspond to the wintering period. For the M_3_, OS and ERJ endmembers were expected to correspond respectively to the winter and to the late summer/autumn periods.

### Solution analysis

For the solution data, sampling and isotope analysis was done in a prior study and is detailed in Britton et al.^23^. To summarise, the second and third molars (M_2_ and M_3_) were extracted from the mandibles, brush-cleaned with water and left to dry overnight. The whole teeth were mechanically abraded to remove surficial enamel. Sampling was done on the buccal face of the anterior loph as it presented a thicker enamel, with the face removed and cleaned from adhering dentine using a tungsten carbide burr. Enamel faces were then marked for horizontal sectioning at ∼ 1.5 mm intervals, ultrasonicated in deionize water (DI H_2_O, 18.3 MΩ) and dried, before being cut into strips using diamond-coated superfine circular drill bits. Samples were then individually ultrasonicated in DI H_2_O, dried and split longitudinally with ∼ 5 mg of enamel being reserved for ^87^Sr/^86^Sr solution analysis and the remainder being retained for carbon and oxygen isotope analysis. In that study, sections were numerically assigned from the ERJ to the OS (M_2_-1, M_2_-2, M_2_-3,…).

Strontium was isolated from enamel in clean laboratory facilities at the Department of Human Evolution, Max Planck Institute for Evolutionary Anthropology (MPI-EVA), Leipzig, Germany using a modified version of the method from Deniel and Pin^28^ described in detail in Copeland et al.^34^. The following description of the analytical procedure is reproduced from Britton et al.^23^, and is also presented there in full. The ∼ 5 mg samples were dissolved in 1 ml 14.3 M high purity HNO_3_ then evaporated to dryness. The obtained residue was then re-dissolved in 1 ml 3 M HNO_3_ before loading into pre-conditioned columns containing Sr Resin (Eichrom Technologies, Lisle, IL, USA), being passed through three times. Strontium was then eluted using ultrapure deionized water (18.2 MΩ), dried and re-dissolved in 3% HNO_3_ and analysis of ^87^Sr/^86^Sr ratios was undertaken using a Thermo Fisher Neptune™ (MC-ICP-MS). All the acids solutions used in the procedure were purified through a PicoTrace double-distilled sub-boiling distillation system. The subsequent ^87^Sr/^86^Sr measurements on standards and samples were corrected for interferences from krypton (Kr) and rubidium (Rb) and normalized for instrumental mass bias to ^88^Sr/^86^Sr = 8.375209 (exponential law). Analysis of the international strontium isotope standard NIST SRM987 (National Institute of Standards and Technology, Gaithersburg, USA) during each analytical session was used for external normalisation of data (long-term ^87^Sr/^86^Sr value = 0.710273 ± 0.000033 (2*σ*) (*n* = 97)). All ^87^Sr/^86^Sr values reported here were adjusted so SRM987 = 0.710240^72^, involving a data correction factor of − 0.00002. Strontium concentrations of the enamel samples were determined using the method described in^34^, which is accurate to within ± 31 ppm.

### LA-MC-ICP-MS analysis

Enamel samples intended for analysis via LA-MC-ICP-MS were cut from whole teeth using diamond-coated superfine circular drill bits as sections encompassing the whole height of the crown just below the ERJ to the OS. The use of diamond coated drill bits serves to produce both a flat and partially polished cut surface. Resulting sections were then ultrasonicated in DI H_2_O for five minutes and then dried in an air extraction hood at room temperature overnight.

Strontium isotope data were measured from the samples using a Coherent GeoLas 193 nm excimer laser ablation (LA) system coupled to a Thermo-Fisher Neptune™ (MC-ICP-MS) located at the MicroAnalysis Facility in the CREAIT Network, Memorial University of Newfoundland, Canada. Up to four samples at a time were mounted planar (laying flat with enamel and adjacent dentine facing up and exposing the full height of the crown in cross-section) on tack in the laser’s sample chamber (Fig. S13). The analyses were done as a continuous laser line scan in the enamel from OS to ERJ, with a cycle of measurement done approximately every 21 µm along the pathway. In each instance, the sampling pathway was pre-ablated prior to ablation and measurement to removal surficial enamel.

Typical operating parameters of the LA-MC-ICP-MS are given in Table S2. The parameters were tuned to maximize signal intensity while minimizing oxide formation by ablating a synthetic glass containing low rare earth elements (LREE-doped glass^73^) and monitoring for the formation of LREE-oxides as a general proxy for oxide formation. Nitrogen was added to the Ar sample make-up gas to break down/suppress interferences^74^. The potential polyatomic interferences on the strontium isotopes were managed by this combination of low oxide tuning and nitrogen addition. Doubly charged REEs were assumed to be a negligible interference since ^89^Y was not present in any significant amount. The signal intensity of ^88^Sr ranged from 3 to 8 V for the samples. First, the measured values were corrected for Kr using a gas blank subtraction. Second, ^87^Sr was corrected for Rb assuming the natural abundance of ^87^Rb/^85^Rb and a correction for mass bias using the mass bias of Sr. Since Rb/Sr was low in these samples, this model of Rb correction was sufficient. Finally, the ^87^Sr/^86^Sr values were normalized for instrumental mass bias to ^88^Sr/^86^Sr = 8.375209 (exponential law). ^84^Sr/^86^Sr was also calculated for each sample (average = 0.0562 ± 0.004 SD) and compared to the invariant ^84^Sr/^86^Sr = 0.0565 as a quality control check. An in-house standard (SW1—modern sperm whale enamel) was also measured in each session. The average ^87^Sr/^86^Sr = 0.70922 ± 0.00006 SD (n = 7) was compared to ^87^Sr/^86^Sr = 0.7092 for present-day seawater as a check for accuracy of the laser data.

### Comparison between solution and LA-MC-ICP-MS profiles

For each tooth we calculated from the raw LA-MC-ICP-MS ^87^Sr/^86^Sr values a running average using a window of 20 data points to obtain the corresponding LA-MC-ICP-MS profile. We then extracted from the solution and LA-MC-ICP-MS profiles the minimum, maximum and median ^87^Sr/^86^Sr values. We also considered endmember ^87^Sr/^86^Sr values, i.e., the samples closest to the OS and to the ERJ in each profile^26,35^. Due to the low number of samples, we conducted non-parametric Wilcoxon signed-rank test to compare the different metrics assessed from solution and LA-MC-ICP-MS ^87^Sr/^86^Sr intra-tooth profiles.

### Geographic assignment

For the 5 individuals, we performed geographic assignment of the endmember ^87^Sr/^86^Sr values of both teeth analysed via the two different methods (solution and LA-MC-ICP-MS) using the assignR package^53^ in R software (v4.2.1)^54^. The assignR package uses Bayesian spatial assignment to infer the geographic origin of samples from ^87^Sr/^86^Sr isoscape. We used the global bioavailable ^87^Sr/^86^Sr isoscape published by Bataille et al.^14^ and the associated spatial error raster. This isoscape is based on extensive collection of field sampling of plants, soils and water, the data being drawn from a variety of studies published over the last decade (see Bataille et al.^14^ for the list of references). Endmembers were treated as samples of unknown origin, and we generated a posterior probability surface, rescaled to 1, for each sample. For both solution and LA-MC-ICP-MS values of a given endmember, we extracted the 20% of the posterior probability surface with the highest probability of origin and calculated the overlap between the two assignment surfaces. Finally, we used the oddRatio tool from assignR to compare with odd ratio the posterior probabilities between summer and winter ranges and assessed from which seasonal ranges endmembers were more likely to originate from (odd ratio = probability of assignment summer range/probability of assignment winter range). Shapefiles of summer and winter ranges were obtained from the Alaska Center for Conservation Science^55^ and are based on the telemetric survey of the herd by the Alaska Department of Fish and Game between 2004 and 2014.

## Supplementary Information

Supplementary Information 1.

Supplementary Information 2.

Supplementary Information 3.

## Data Availability

Isotope data described in the manuscript and R code are provided in the Supplementary Information ESM2 and ESM3 respectively. Strontium isoscape and associated error map are available from Bataille et al.^14^.
