# Supplementary material for: Comparison between strip sampling and laser ablation methods to infer seasonal movements from intra-tooth strontium isotopes profiles in migratory caribou
Source: Sci Rep. 2023 Mar 3;13:3621. doi: 10.1038/s41598-023-30222-w (PMC9984400; doi:10.1038/s41598-023-30222-w)
Supplement: Supplementary file 1 — Supplementary Information 1. [file 41598_2023_30222_MOESM1_ESM.docx]

**Comparison between strip sampling and laser ablation methods to infer seasonal movements from intra-tooth strontium isotopes profiles in migratory caribou**

Mael Le Corre^1^*, Vaughan Grimes^2,3^, Rebecca Lam^4^ and Kate Britton^1^*?

^1^Department of Archaeology, University of Aberdeen, Aberdeen, AB252SU, United Kingdom

^2^Department of Archaeology, Memorial University of Newfoundland, St. John's, NL A1C 5S7, Canada

^3^Department of Earth Sciences, Memorial University of Newfoundland, St. John’s, NL A1C 5S7, Canada

^4^CREAIT Network, Memorial University of Newfoundland, St. John's, NL A1C 5S7, Canada

*Corresponding authors: MLC and KB

Emails:

MLC: mael.lecorre@abdn.ac.uk

KB: k.britton@abdn.ac.uk


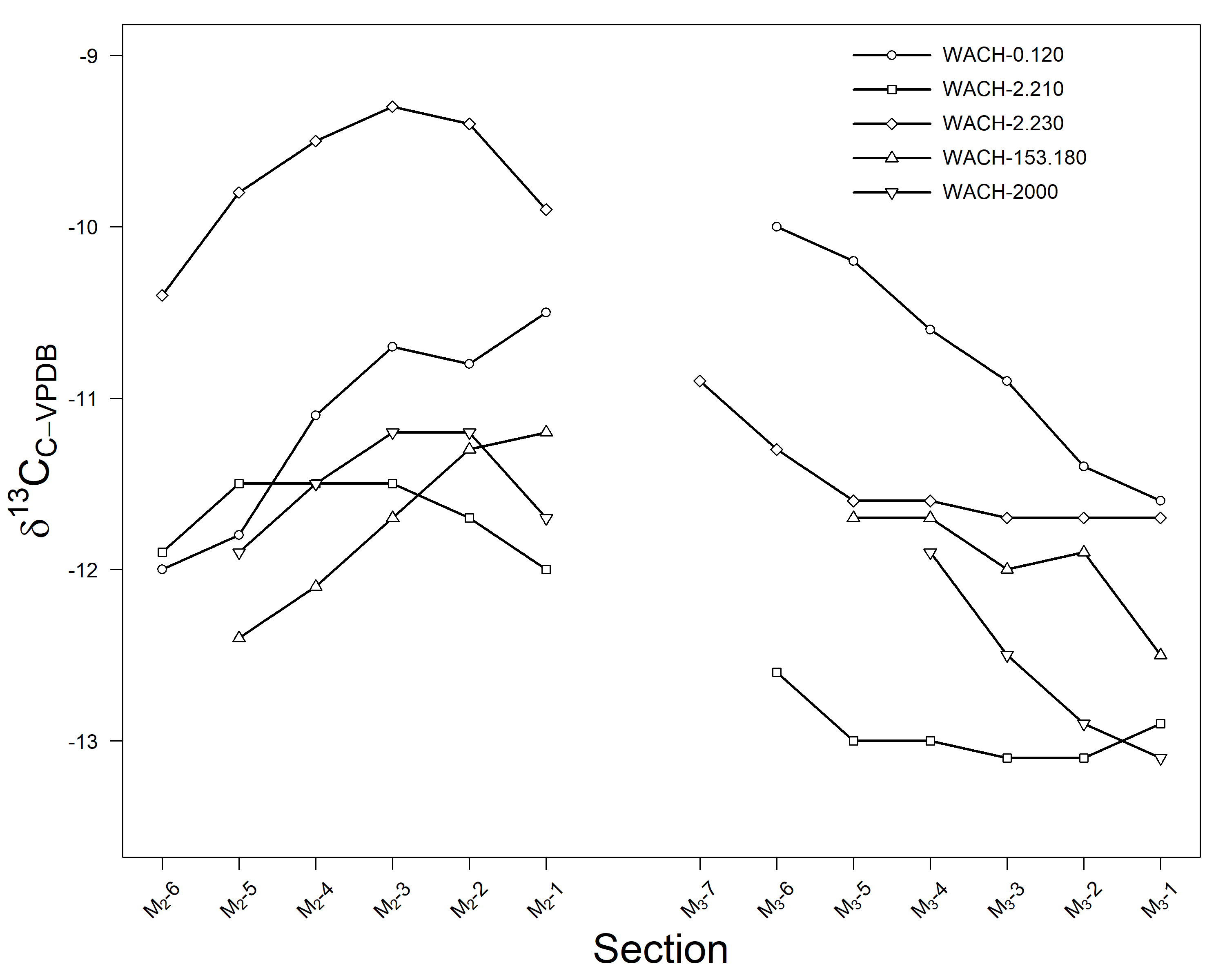
Figure S1. Carbon isotope (*ẟ*^13^C_V-PDB_) intra-tooth enamel profiles from the second and third molars of 5 caribou from the Western Arctic herd, Alaska. Reproduced from Britton^1^.


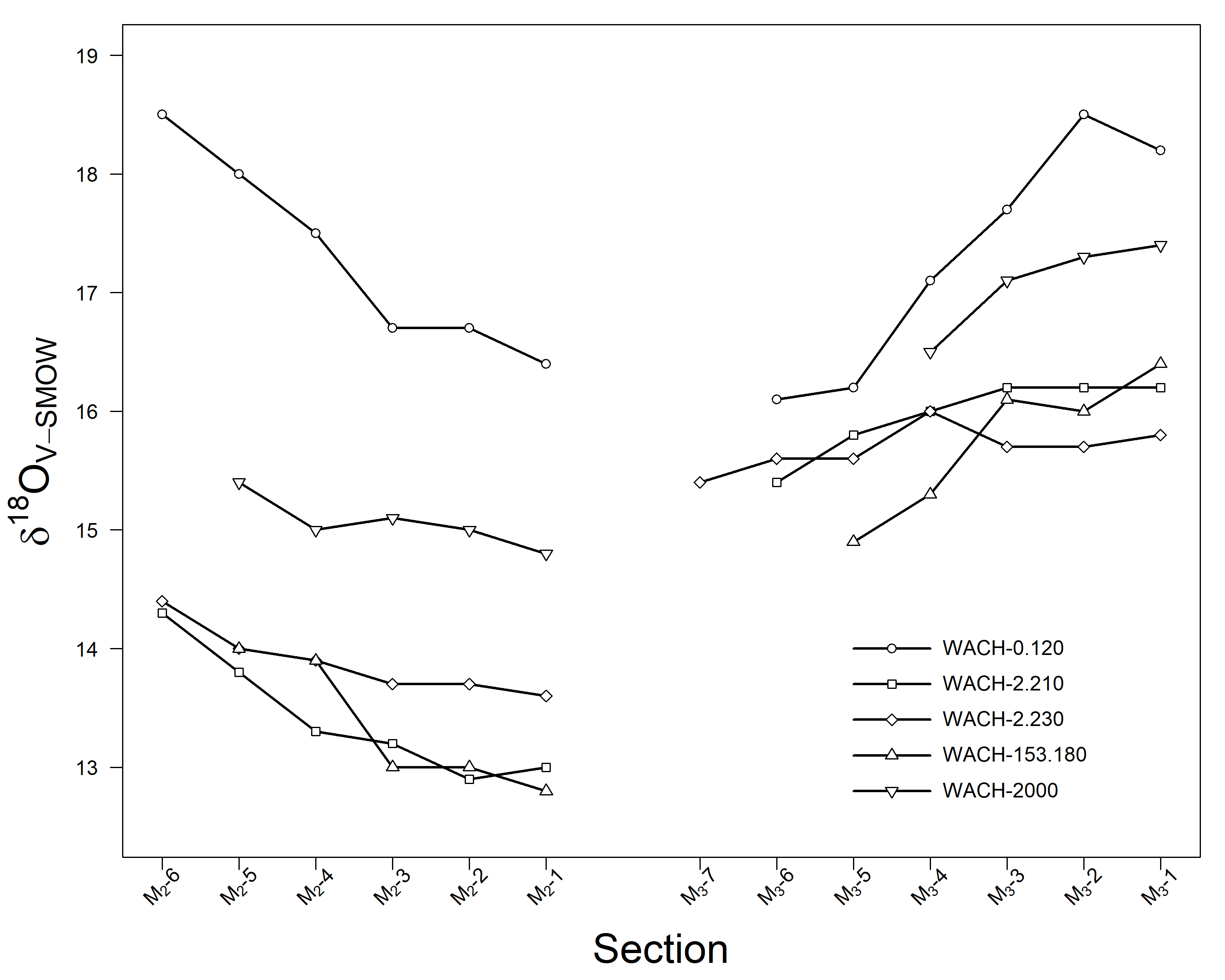
Figure S2. Oxygen isotope (*ẟ*^18^O_V-SMOW_) intra-tooth enamel profiles from the second and third molars of 5 caribou from the Western Arctic herd, Alaska. Reproduced from Britton et al.^2^.


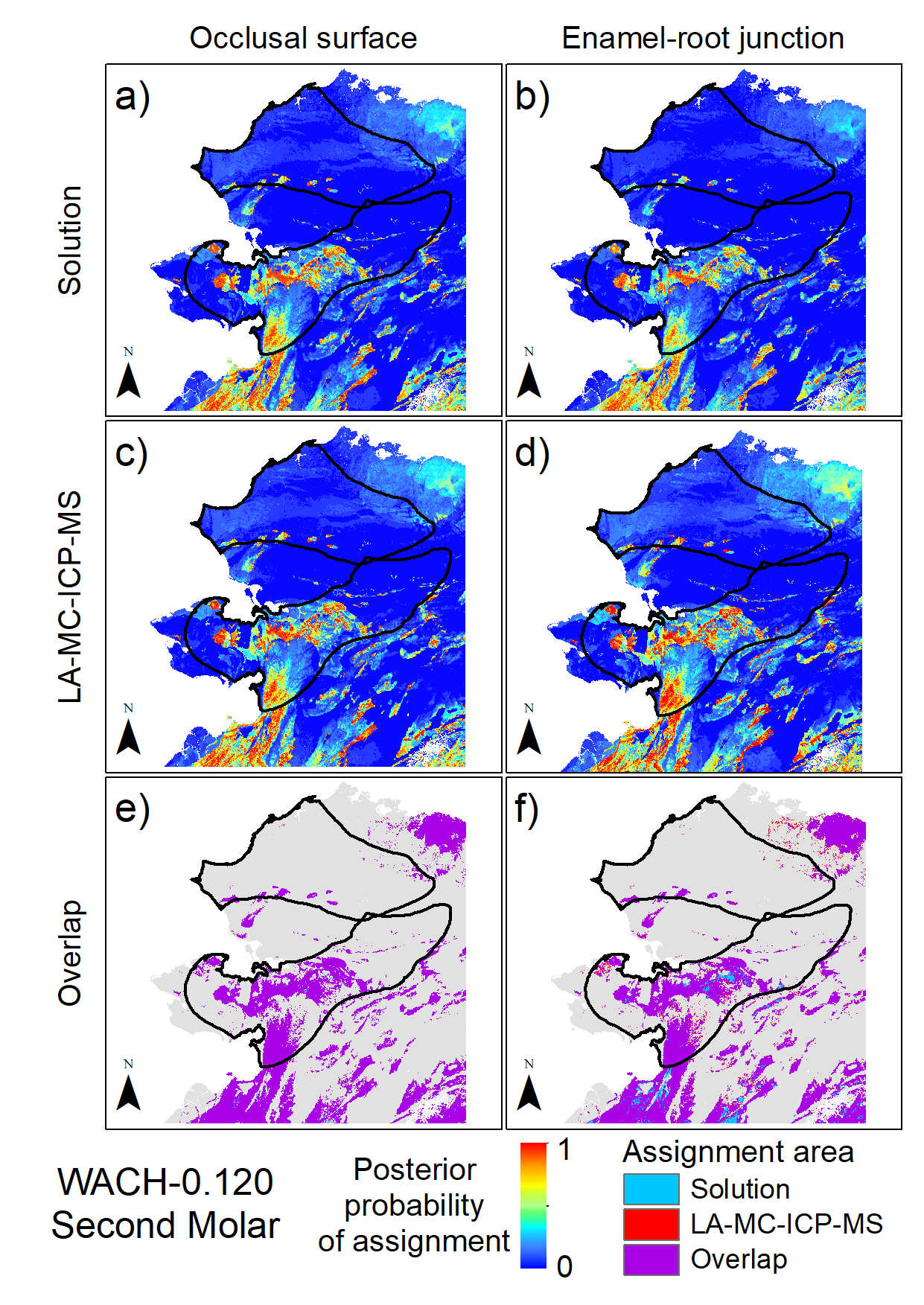


Figure S3. Spatial assignment of the endmembers of the ^87^Sr/^86^Sr intra-tooth profile from the second molar of caribou WACH-0.120. Left and right columns display the spatial assignment for the sample taken close to the occlusal surface and close to the enamel-root junction, respectively. Posterior probably of assignment are shown a) and b) for the values obtained from the solution, c) and d) for the values obtained from the LA-MC-ICP-MS analysis. e) and f) show the overlap between the areas corresponding to 20% of the map with the highest posterior probability assessed from the two methods of ^87^Sr/^86^Sr analysis. Summer (north) and winter (south) ranges are delineated in black. The maps were generated in R (v4.2.1) and formatted using ArcGIS 10.5.


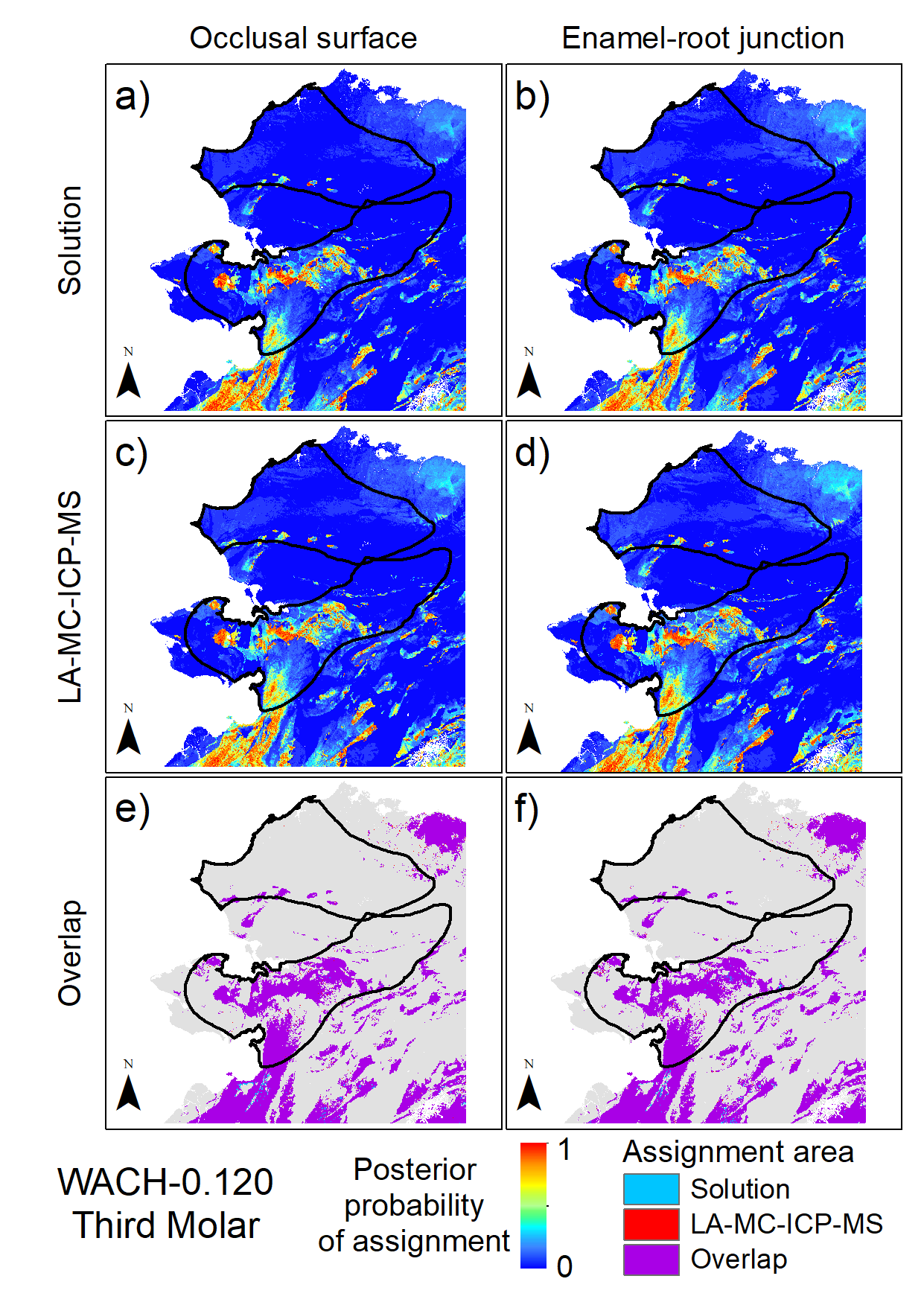


Figure S4. Spatial assignment of the endmembers of the ^87^Sr/^86^Sr intra-tooth profile from the third molar of caribou WACH-0.120. Left and right columns display the spatial assignment for the sample taken close to the occlusal surface and close to the enamel-root junction, respectively. Posterior probably of assignment are shown a) and b) for the values obtained from the solution, c) and d) for the values obtained from the LA-MC-ICP-MS analysis. e) and f) show the overlap between the areas corresponding to 20% of the map with the highest posterior probability assessed from the two methods of ^87^Sr/^86^Sr analysis. Summer (north) and winter (south) ranges are delineated in black. The maps were generated in R (v4.2.1) and formatted using ArcGIS 10.5.


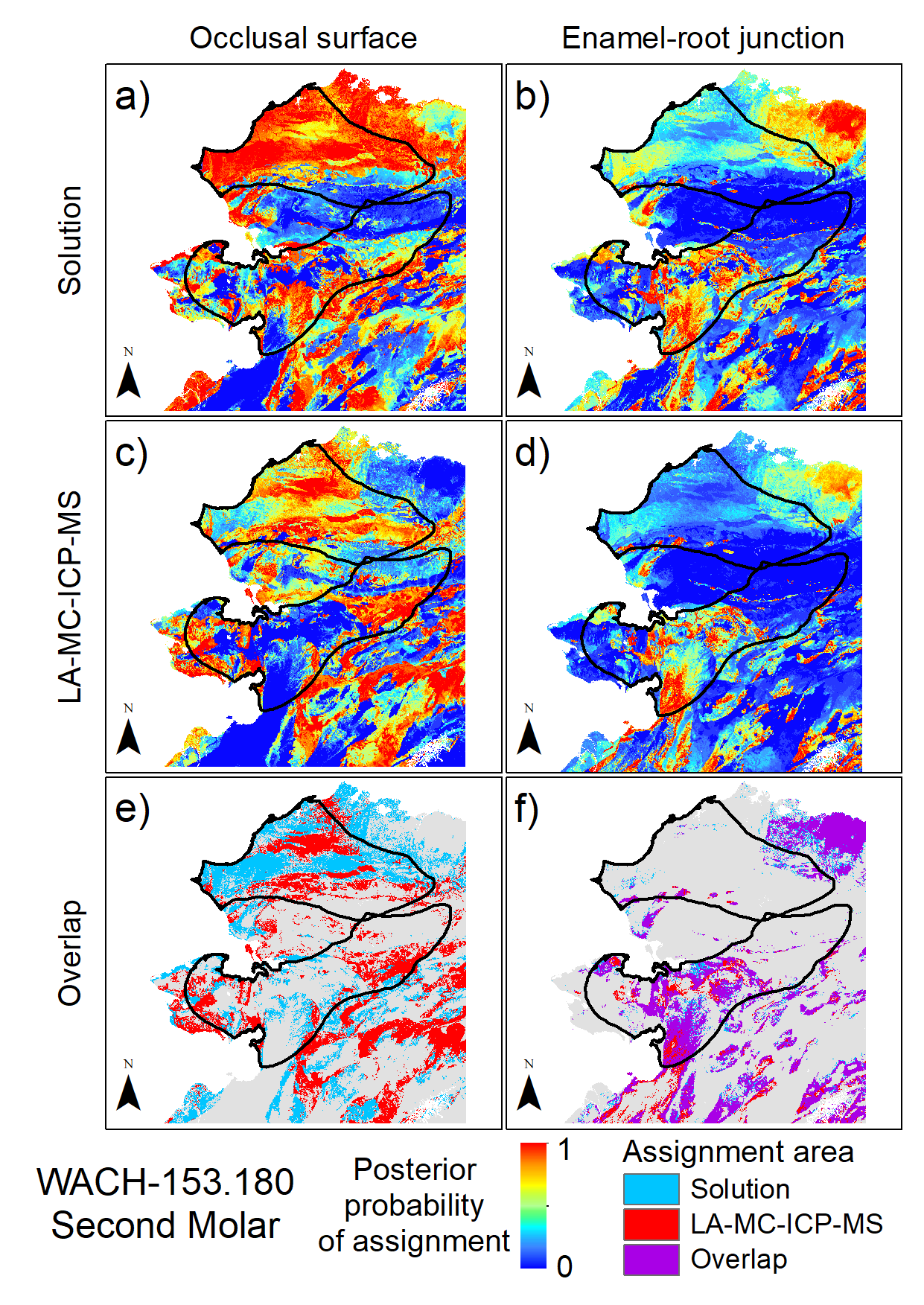


Figure S5. Spatial assignment of the endmembers of the ^87^Sr/^86^Sr intra-tooth profile from the second molar of caribou WACH-153.180. Left and right columns display the spatial assignment for the sample taken close to the occlusal surface and close to the enamel-root junction, respectively. Posterior probably of assignment are shown a) and b) for the values obtained from the solution, c) and d) for the values obtained from the LA-MC-ICP-MS analysis. e) and f) show the overlap between the areas corresponding to 20% of the map with the highest posterior probability assessed from the two methods of ^87^Sr/^86^Sr analysis. Summer (north) and winter (south) ranges are delineated in black. The maps were generated in R (v4.2.1) and formatted using ArcGIS 10.5.


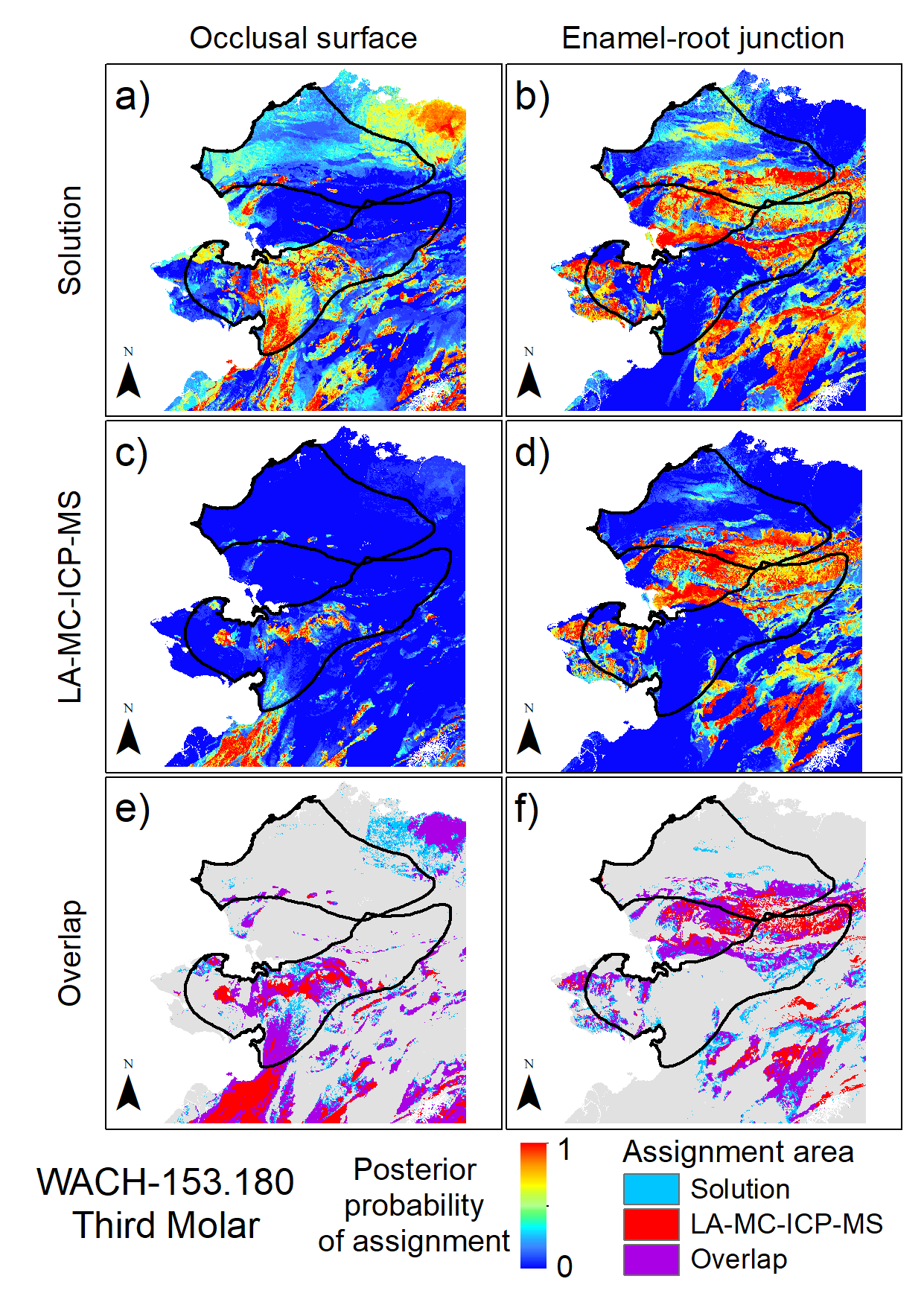


Figure S6. Spatial assignment of the endmembers of the ^87^Sr/^86^Sr intra-tooth profile from the third molar of caribou WACH-153.180. Left and right columns display the spatial assignment for the sample taken close to the occlusal surface and close to the enamel-root junction, respectively. Posterior probably of assignment are shown a) and b) for the values obtained from the solution, c) and d) for the values obtained from the LA-MC-ICP-MS analysis. e) and f) show the overlap between the areas corresponding to 20% of the map with the highest posterior probability assessed from the two methods of ^87^Sr/^86^Sr analysis. Summer (north) and winter (south) ranges are delineated in black. The maps were generated in R (v4.2.1) and formatted using ArcGIS 10.5.


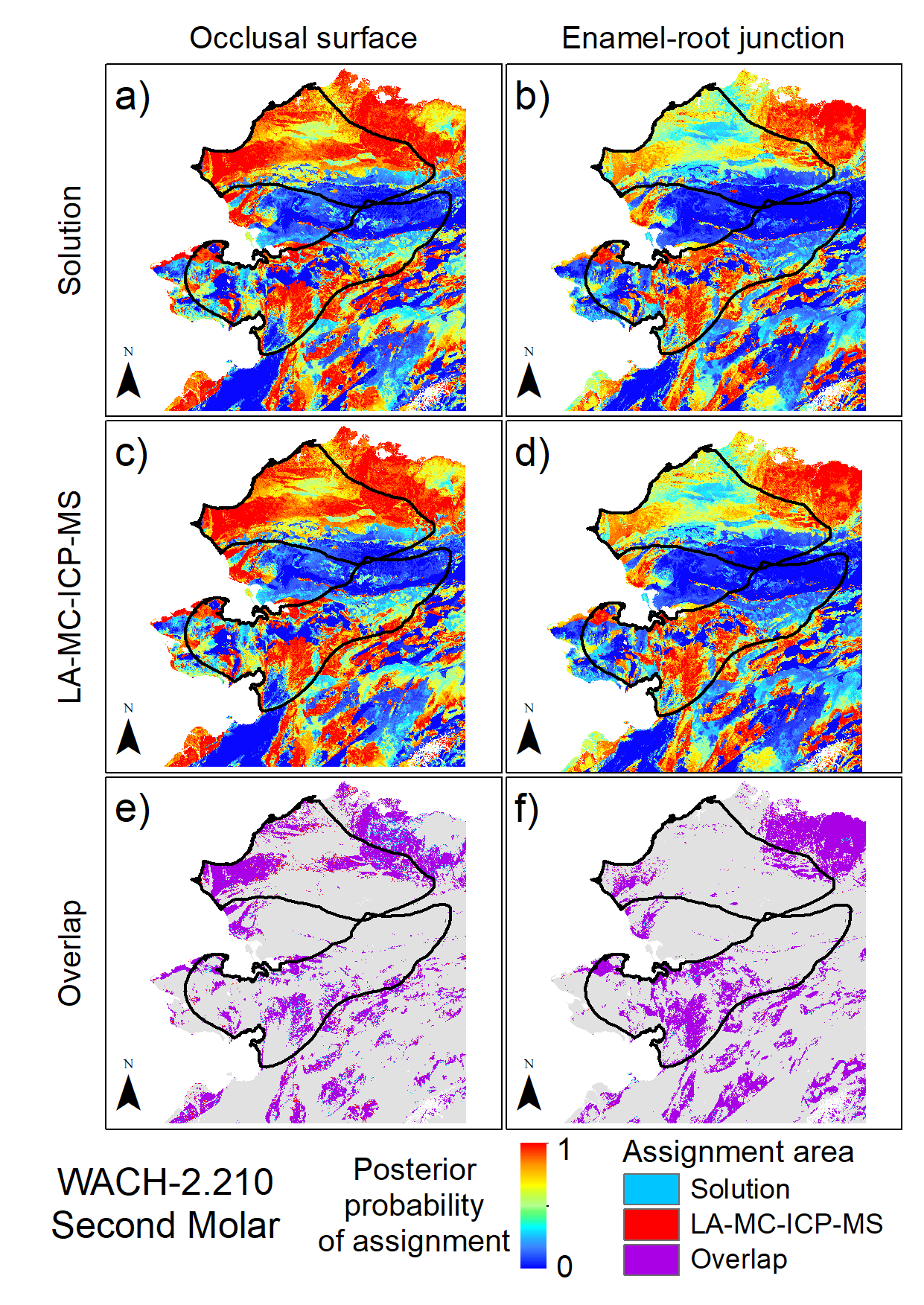


Figure S7. Spatial assignment of the endmembers of the ^87^Sr/^86^Sr intra-tooth profile from the second molar of caribou WACH-2.210. Left and right columns display the spatial assignment for the sample taken close to the occlusal surface and close to the enamel-root junction, respectively. Posterior probably of assignment are shown a) and b) for the values obtained from the solution, c) and d) for the values obtained from the LA-MC-ICP-MS analysis. e) and f) show the overlap between the areas corresponding to 20% of the map with the highest posterior probability assessed from the two methods of ^87^Sr/^86^Sr analysis. Summer (north) and winter (south) ranges are delineated in black. The maps were generated in R (v4.2.1) and formatted using ArcGIS 10.5.


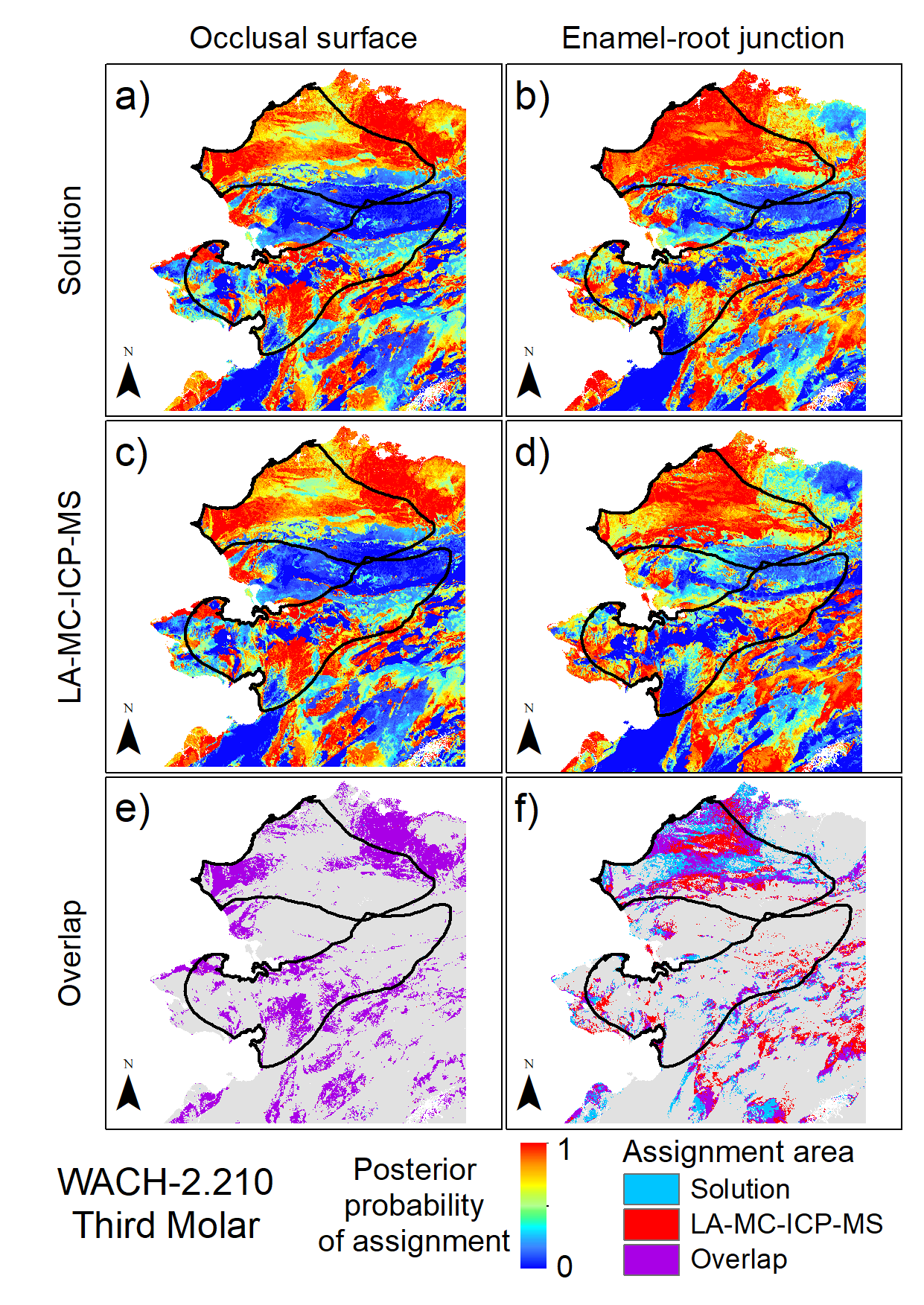


Figure S8. Spatial assignment of the endmembers of the ^87^Sr/^86^Sr intra-tooth profile from the third molar of caribou WACH-2.210. Left and right columns display the spatial assignment for the sample taken close to the occlusal surface and close to the enamel-root junction, respectively. Posterior probably of assignment are shown a) and b) for the values obtained from the solution, c) and d) for the values obtained from the LA-MC-ICP-MS analysis. e) and f) show the overlap between the areas corresponding to 20% of the map with the highest posterior probability assessed from the two methods of ^87^Sr/^86^Sr analysis. Summer (north) and winter (south) ranges are delineated in black. The maps were generated in R (v4.2.1) and formatted using ArcGIS 10.5.


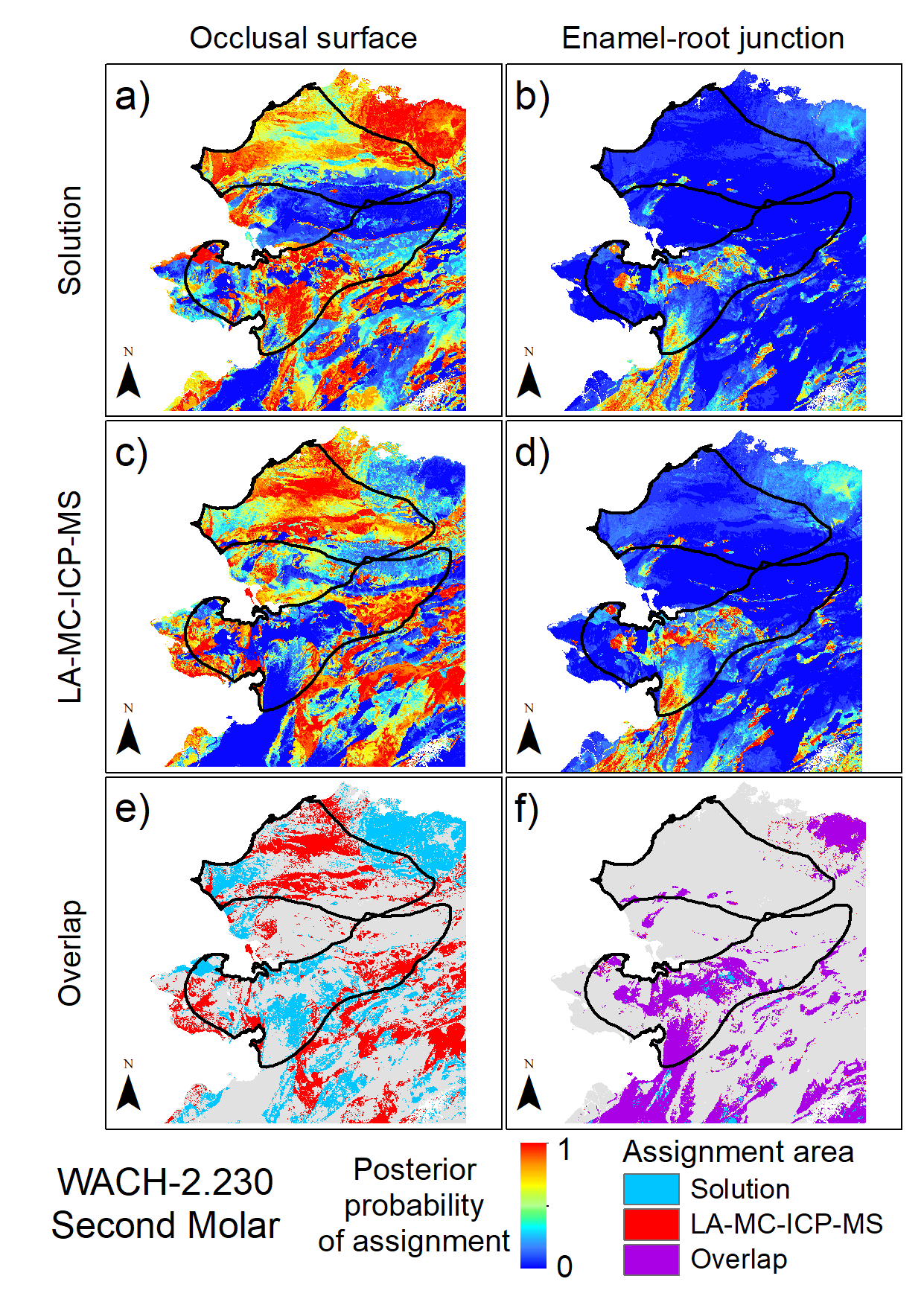


Figure S9. Spatial assignment of the endmembers of the ^87^Sr/^86^Sr intra-tooth profile from the second molar of caribou WACH-2.230. Left and right columns display the spatial assignment for the sample taken close to the occlusal surface and close to the enamel-root junction, respectively. Posterior probably of assignment are shown a) and b) for the values obtained from the solution, c) and d) for the values obtained from the LA-MC-ICP-MS analysis. e) and f) show the overlap between the areas corresponding to 20% of the map with the highest posterior probability assessed from the two methods of ^87^Sr/^86^Sr analysis. Summer (north) and winter (south) ranges are delineated in black. The maps were generated in R (v4.2.1) and formatted using ArcGIS 10.5.


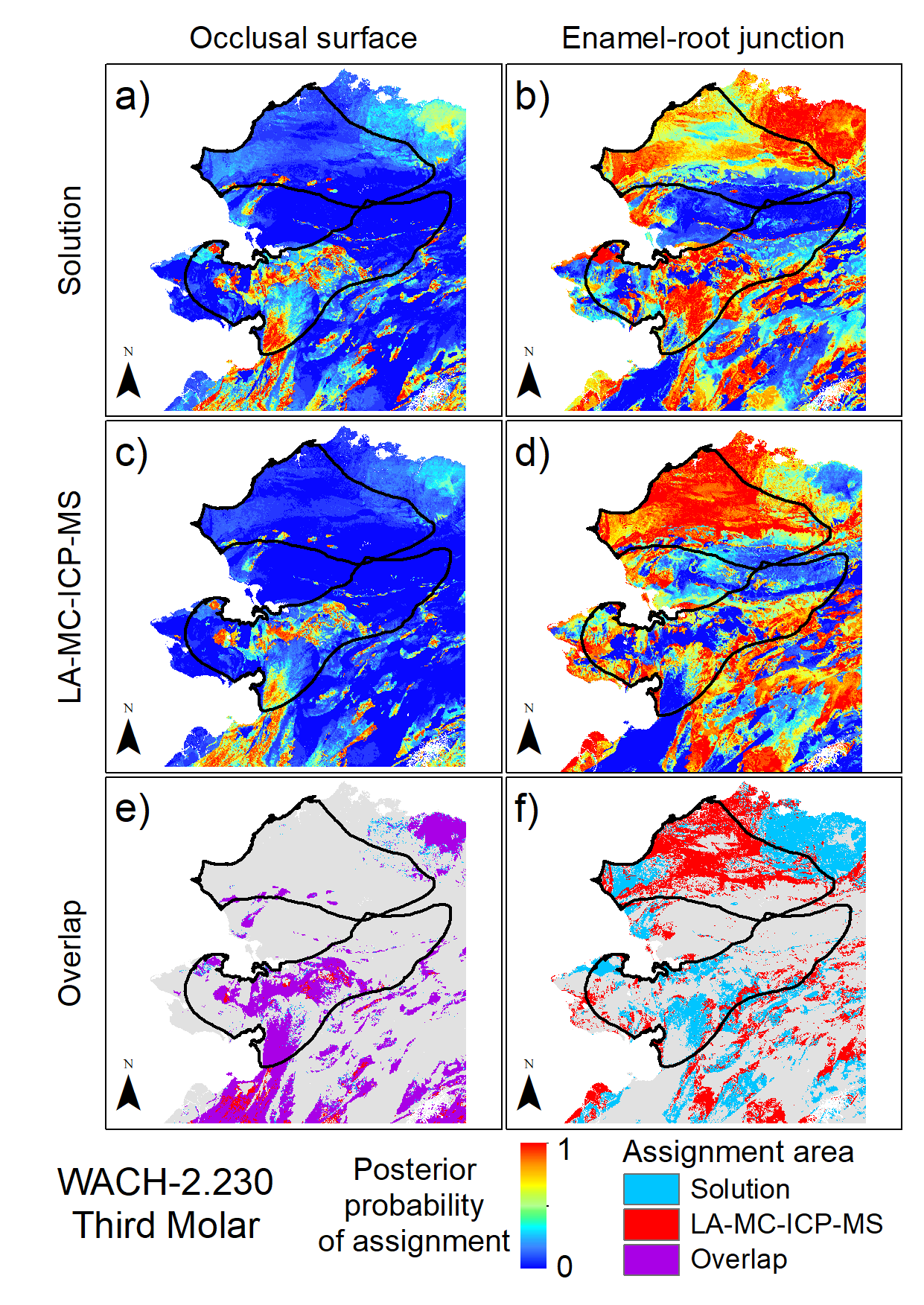


Figure S10. Spatial assignment of the endmembers of the ^87^Sr/^86^Sr intra-tooth profile from the third molar of caribou WACH-2.230. Left and right columns display the spatial assignment for the sample taken close to the occlusal surface and close to the enamel-root junction, respectively. Posterior probably of assignment are shown a) and b) for the values obtained from the solution, c) and d) for the values obtained from the LA-MC-ICP-MS analysis. e) and f) show the overlap between the areas corresponding to 20% of the map with the highest posterior probability assessed from the two methods of ^87^Sr/^86^Sr analysis. Summer (north) and winter (south) ranges are delineated in black. The maps were generated in R (v4.2.1) and formatted using ArcGIS 10.5.


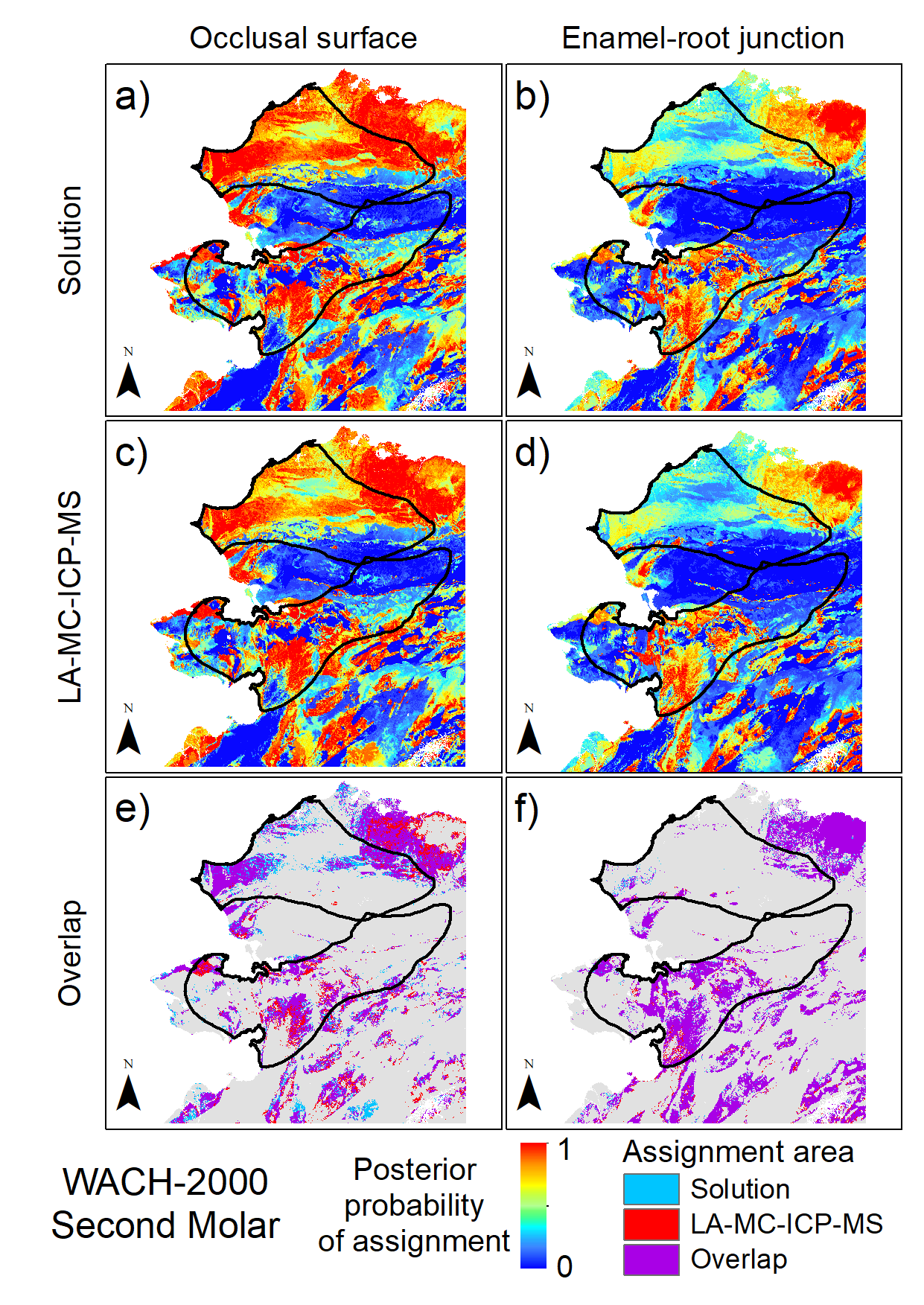


Figure S11. Spatial assignment of the endmembers of the ^87^Sr/^86^Sr intra-tooth profile from the second molar of caribou WACH-2000. Left and right columns display the spatial assignment for the sample taken close to the occlusal surface and close to the enamel-root junction, respectively. Posterior probably of assignment are shown a) and b) for the values obtained from the solution, c) and d) for the values obtained from the LA-MC-ICP-MS analysis. e) and f) show the overlap between the areas corresponding to 20% of the map with the highest posterior probability assessed from the two methods of ^87^Sr/^86^Sr analysis. Summer (north) and winter (south) ranges are delineated in black. The maps were generated in R (v4.2.1) and formatted using ArcGIS 10.5.


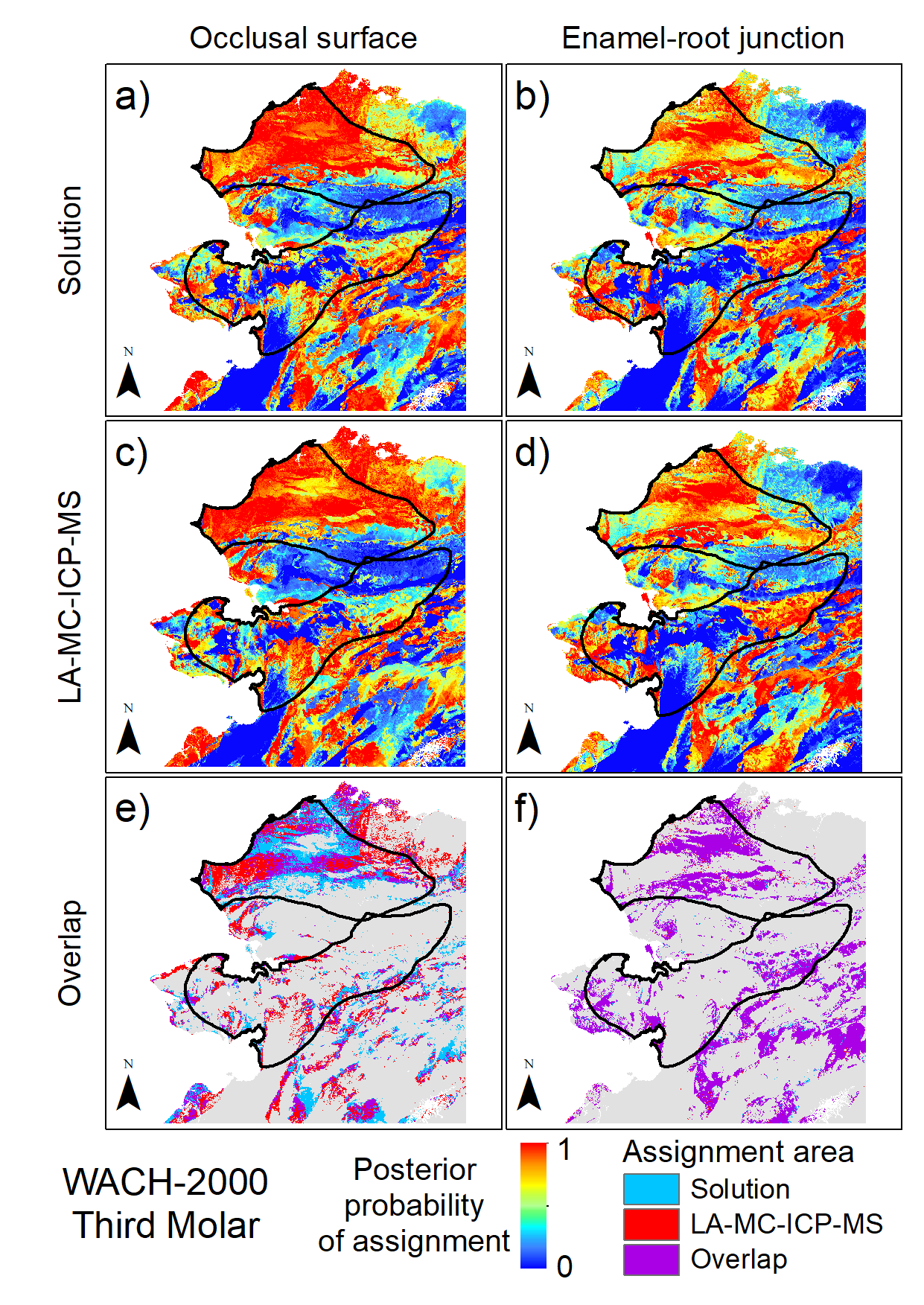


Figure S12. Spatial assignment of the endmembers of the ^87^Sr/^86^Sr intra-tooth profile from the third molar of caribou WACH-2000. Left and right columns display the spatial assignment for the sample taken close to the occlusal surface and close to the enamel-root junction, respectively. Posterior probably of assignment are shown a) and b) for the values obtained from the solution, c) and d) for the values obtained from the LA-MC-ICP-MS analysis. e) and f) show the overlap between the areas corresponding to 20% of the map with the highest posterior probability assessed from the two methods of ^87^Sr/^86^Sr analysis. Summer (north) and winter (south) ranges are delineated in black. The maps were generated in R (v4.2.1) and formatted using ArcGIS 10.5.


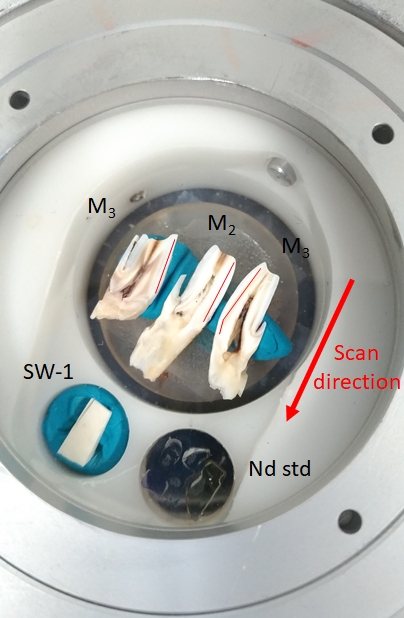


Figure S13. Example of the analytical set up for the caribou teeth in the Coherent GeoLas 193 nm excimer (LA) sample chamber, coupled with the Thermo-Fisher Neptune™ MC-ICP-MS. Exemplar laser track routes are indicated in red.

Table S1. Mandibles and tooth surfaces sampled for the solution analysis and the LA-MC-ICP-MS analysis. Crown height (in mm) is provided for teeth analysed with LA-MC-ICP-MS method. This information is unknown for teeth analysed with solution method. Instead, the number of sections sampled in the tooth (∼1.5 mm) is provided as an indication of the crown height.

|  |  | Solution | | | LA-MC-ICP-MS | | |  |
| --- | --- | --- | --- | --- | --- | --- | --- | --- |
| individual | molar | mandible sampled | surface sampled | number of sections | mandible sampled | surface sampled | crown height (mm) |  |
|  |  |  |  |  |  |  |  |  |
| WACH-0.120 | M_2_ | Left | buccal | 6 | Left | lingual | 8.19 |  |
| WACH-0.120 | M_3_ | Left | buccal | 6 | Left | lingual | 10.09 |  |
| WACH-153.180 | M_2_ | Right | buccal | 5 | Left | lingual | 10.94 |  |
| WACH-153.180 | M_3_ | Right | buccal | 5 | Left | lingual | 10.19 |  |
| WACH-2.210 | M_2_ | Left | buccal | 6 | Right | lingual | 10.66 |  |
| WACH-2.210 | M_3_ | Left | buccal | 6 | Right | lingual | 9.32 |  |
| WACH-2.230 | M_2_ | Left | buccal | 6 | Left | lingual | 8.52 |  |
| WACH-2.230 | M_3_ | Left | buccal | 7 | Left | lingual | 10.92 |  |
| WACH-2000 | M_2_ | Right | buccal | 5 | Right | lingual | 7.12 |  |
| WACH-2000 | M_3_ | Right | buccal | 4 | Right | lingual | 8.49 |  |

Table S2: LA-MC-ICP-MS operating parameters.

| **MC-ICP-MS** | | |
| --- | --- | --- |
| RF Power | 1200 W | |
| Resolution | Low | |
| Cool/Plasma gas flow rate (Ar) | 16 l/min | |
| Auxiliary gas flow rate (Ar) | 1.0 l/min | |
| Sample makeup flow rate (Ar) | 0.7 l/min | |
| Additional gas flow rate (N_2_) | 0.015 – 0.020 l/min | |
| Masses in Faraday Cups | ^82^Kr, ^83^Kr, ^84^Sr(+Kr), ^85^Rb, ^86^Sr(+Kr), ^87^Sr(+Rb), ^88^Sr, ^89^Y | |
| Integration time | 1.049 s/cycle | |
| Number of measurements | 400-700 cycles | |
| **Laser** | | |
| Laser cell carrier gas flow rate (He) | 1.0 l/min | |
|  | *Preablation* | *Ablation* |
| Laser energy | 2 J/cm^2^ | 10 J/cm^2^ |
| Repetition rate | 2 Hz | 10 Hz |
| Spot size | 158 µm | 158 µm |
| Scan rate | 100 µm/s | 20 µm/s |

Electronic Supplementary Material ESM2. Solution and LA-MC-ICP-MS ^87^Sr/^86^Sr intra-tooth data.

Excel file with the ^87^Sr/^86^Sr intra-tooth data of 5 caribou from the Western Arctic Herd obtained using solution analysis (“solution_sr” sheet, generated by Britton et al.^2^) and using LA-MC-ICP-MS analysis (“LA-MC-ICP-MS_sr” sheet).

Electronic Supplementary Material ESM3. R code.

R code used to produce Fig.1 and performed the geographic assignment of the endmembers of the ^87^Sr/^86^Sr intra-tooth profiles from 5 caribou generated using solution and LA-MC-ICP-MS analyses.

References

1. Britton, K. Multi-isotope analysis and the reconstruction of prey species palaeomigrations and palaeoecology. (Durham University, 2010).

2. Britton, K., Grimes, V., Dau, J. & Richards, M. P. Reconstructing faunal migrations using intra-tooth sampling and strontium and oxygen isotope analyses: a case study of modern caribou (*Rangifer tarandus granti* ). *J Archaeol Sci* **36**, 1163–1172 (2009).
